# *In Situ* Raman Hyperspectral Analysis of Microbial Colonies for Secondary Metabolites Screening

**DOI:** 10.1021/acs.analchem.4c02906

**Published:** 2024-08-31

**Authors:** Shunnosuke Suwa, Masahiro Ando, Takuji Nakashima, Shumpei Horii, Toyoaki Anai, Haruko Takeyama

**Affiliations:** † Department of Advanced Science Engineering, Graduate School of Advanced Science and Engineering, Waseda University, 3-4-1 Okubo, Shinjuku-Ku, Tokyo 169-8555, Japan; ‡ Computational Bio Big-Data Open Innovation Laboratory (CBBD-OIL), National Institute of Advanced Industrial Science and Technology, 3-4-1 Okubo, Shinjuku-Ku, Tokyo 169-8555, Japan; § Research Organization for Nano and Life Innovation, Waseda University, 513 Wasedatsurumaki-Cho, Shinjuku-Ku, Tokyo 162-0041, Japan; ∥ Faculty of Agriculture, Kyushu University, 744 Motooka, Nishi-ku, Fukuoka, Fukuoka 819-0395 Japan; ⊥ Institute for Advanced Research of Biosystem Dynamics, Graduate School of Advanced Science and Engineering, Waseda Research Institute for Science and Engineering, Waseda University, 3-4-1 Okubo, Shinjuku-Ku, Tokyo 169-8555, Japan; # Department of Life Science and Medical Bioscience, Graduate School of Advanced Science and Engineering, Waseda University, 2-2 Wakamatsu-Cho, Shinjuku-Ku, Tokyo 162-8480, Japan

## Abstract

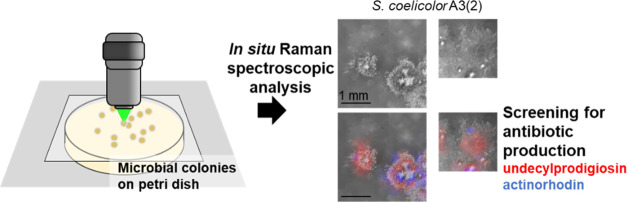

Since the discovery of penicillin, a vast array of microbial antibiotics has been identified and applied in the medical field. Globally, the search for drug candidates *via* microbial screening is ongoing. Traditional screening methods, however, are time-consuming and require labor-intensive sample processing, significantly reducing throughput. This research introduces a Raman spectroscopy-based screening system tailored to the *in situ* analysis of microbial colonies on solid culture media. Employing multivariate curve resolution-alternating least-squares (MCR-ALS) for spectral decomposition, our approach reveals the production of secondary metabolites at the single colony level. We enhanced the microbial culture method, enabling direct, high signal-to-noise (S/N) ratio Raman spectroscopic measurements of colonies of *Escherichia coli* and actinomycetes species. Through semisupervised MCR analysis using the known spectra of actinorhodin and undecylprodigiosin as references, we accurately assessed the production of these compounds by *Streptomyces coelicolor* A3(2). Furthermore, we herein successfully detected the production of amphotericin B by *Streptomyces nodosus*, even in the absence of prior spectral information. This demonstrates the potential of our technique in the discovery of secondary metabolites. In addition to enabling the detection of the above-mentioned compounds, this analysis revealed the heterogeneity of the spatial distribution of their production in each colony. Our technique makes a significant contribution to the advancement of microbial screening, offering a rapid, efficient alternative to conventional methods and opening avenues for secondary metabolites discovery.

Microbes are known for producing a variety of antibiotic compounds. Currently, approximately 33,000 types of secondary metabolites have been isolated from microbes.^1^ Though secondary metabolites are not required for the normal growth, development, or reproduction of the organism, these compounds often exhibit significant antibiotic properties and are widely converted into drugs in the medical field.^2^ Notable examples include penicillin, discovered in 1928 and commonly used as an antibiotic,^2^ and actinomycin, exhibiting extensive antitumor activity and employed as an anticancer drug.^3^ Actinomycetes constitute a group of microorganisms particularly prolific in producing secondary metabolites with diverse structures. Approximately 40% of existing antibiotic compounds are derived from actinomycetes,^1^ making them a key focus in the search for novel secondary metabolites. Continued efforts toward the discovery of such compounds highlight the importance of screening for new compounds in drug discovery research.

Screening for microbial antibiotics typically involves several stages.^4−6^ Environmental samples, such as soil or seawater, are first collected, from which microbes are then isolated on Petri dishes. Strains are subsequently selected for screening based on colony appearance or researcher expertise. These are then cultured to produce antibiotic compounds, and the culture extracts are assessed to identify fractions with high antibiotic activity. The target compound is isolated using liquid chromatography/mass spectrometry (LC/MS) and identified *via* nuclear magnetic resonance (NMR) spectroscopy. This approach allows for the detection of trace biomolecules and is bolstered by comprehensive LC/MS and NMR databases, making it highly reliable.^7,8^ However, its drawbacks include lengthy culture times and labor-intensive sample processing. Consequently, only a limited number of strains from the thousands available in environmental samples are analyzed, potentially leading researchers to overlook promising strains with differing metabolic profiles despite similar colony appearances.^9^

Recent advancements have facilitated the direct phenotypic analysis of microbial colonies on Petri dishes. Maeda et al. developed a lensless imaging system employing machine learning for rapid microbial identification.^10^ However, while effective in discriminating between microbial species, this system does not reveal molecular information, presenting challenges in the study of unassigned microbial strains. Imaging mass spectrometry (IMS) offers spatial molecular analysis and has been extensively used for metabolite analysis in microbes.^11,12^ However, its primary limitation is sample destruction, precluding the use of the same samples in additional analytical methods. Raman spectroscopy has recently gained prominence in microbial biomolecular analysis.^13^ It allows for the analysis of microbial biomolecules with minimal preparation and no sample destruction and has been used to identify the cellular localization of compounds such as penicillin and avermectin.^14−16^ Additionally, when combined with machine learning, it can effectively discriminate between pathogenic bacteria.^17,18^ However, these measurements still involve a small degree of sample processing. For the rapid measurements and microbial screening, direct biomolecular observation of colonies on Petri dishes are desired.

We propose a new method for screening microbial secondary metabolites exhibiting potential as antibiotics using Raman spectroscopy. This method directly analyzes microbial colonies on agar dishes, detecting secondary metabolite production *via* multivariate Raman spectral analysis. Only one previous study has reported the direct Raman spectroscopic measurement of microbial colonies on Petri dishes;^19^ however, the dish was required to be open due to optical limitations, and the method did not screen for secondary metabolites production, ultimately leading to only the classification of microbial strains based on cluster analysis. We have modified the microbial culture apparatus and optimized the conditions for the *in situ* Raman spectroscopic measurements. Through multivariate curve resolution-alternating least-squares (MCR-ALS) Raman spectral analysis, we have successfully detected secondary metabolite production in actinomycetes. This approach allows for the rapid acquisition of biomolecular information and identification of secondary metabolites production, bypassing the limitations of long-term liquid cultures and the extensive sample processing inherent in conventional screening. Interestingly, this approach allowed for not only metabolite detection but also the observation of heterogeneity in the antibiotic production in each colony in its natural state; this could prove useful in uncovering the complex relationship between the secondary metabolism and mycelial differentiation of *Streptomyces*. Our method can be used to exhaustively investigate various colony types isolated from the environment, potentially accelerating the discovery of new drug candidates.

## Materials and Methods

### Microbial Cultures

We utilized various microbial strains for the experimental work, including *Escherichia coli* K12, *Streptomyces coelicolor* A3(2), *Streptomyces thermocarboxydus*, *Streptomyces nodosus* (NBRC 12895), *Saccharopolyspora* sp., and *Thermomonospora* sp. In addition, *S. coelicolor* A3(2), *S. thermocarboxydus*, *Saccharopolyspora* sp., and *Thermomonospora* sp. were laboratory-isolated microbial strains. These strains were cultured under the following conditions at 27 °C. *E. coli* K12 was cultured in L-Broth (D.W., 100 mL; L-Broth capsules, 1.55 g [MP Biomedicals]; and agar, 1.5 g [Fujifilm Wako Pure Chemical Corp., Osaka, Japan]) overnight. *S. coelicolor* A3(2), *S. thermocarboxydus*, *Saccharopolyspora* sp., and *Thermomonospora* sp. were cultured in a WAP medium (tap water, 100 mL; l-proline, 1.0 g [Fujifilm Wako Pure Chemical Corp., Osaka, Japan]; and agar, 1.5 g). *S. coelicolor* A3(2) was cultured in a WAP medium for up to 10 days to produce undecylprodigiosin and actinorhodin. Under nonproducing conditions, it was cultured in YD medium (tap water, 100 mL; yeast extract, 1.0 g [Kyokuto Pharmaceutical Industrial Co., Ltd., Tokyo, Japan]; d-glucose, 1.0 g [Fujifilm Wako Pure Chemical Corp., Osaka, Japan]; and agar, 1.5 g) for 3 days. *S. nodosus* was cultured in yeast extract starch medium (D.W., 100 mL; yeast extract, 0.2 g; soluble starch, 1.0 g [Fujifilm Wako Pure Chemical Corp., Osaka, Japan]; and agar, 1.0 g) for 4 days to produce amphotericin B (AmB).

### LC/MS Analysis for Secondary Metabolites

The production of actinorhodin and undecylprodigiosin by *S. coelicolor* A3(2) and AmB by *S. nodosus* was confirmed using a Nexera X2 system (Shimadzu Co., Kyoto, Japan) coupled with an LCMS-9030 QTOF mass spectrometer (Shimadzu Co., Kyoto, Japan). Chromatographic separation was performed using a Shim-pack Velox SP-C18 column (2.7 μm, 150 mm × 2.1 mm, Shimadzu Co., Kyoto, Japan) at 40 °C. The gradient elution involved using solvent A (ultrapure water with 0.1% formic acid [both from Fujifilm Wako Pure Chemical Corp., Osaka, Japan]) and solvent B (acetonitrile [Fujifilm Wako Pure Chemical Corp., Osaka, Japan] with 0.1% formic acid) according to the following program: 5% B from 0 to 2 min, 5–100% B from 2 to 12 min, and 100% B from 12 to 27 min. The flow rate was set at 0.2 mL/min, with an injection volume of 2 μL. Ultraviolet (UV) spectra were detected using a photodiode array detector. ESI-TOF-MS was used to record the *m*/*z* region from 100 to 2000 Da for 27 min.

Regarding *S. coelicolor* A3(2), the culture dishes were frozen at −80 °C for secondary metabolite recovery. The culture solution was filtered and extracted from the frozen agar, with 5 M HCl (Fujifilm Wako Pure Chemical Corp., Osaka, Japan) added to slightly acidify the extract. The aqueous solution was then treated with ethyl acetate (EtOAc; Fujifilm Wako Pure Chemical Corp., Osaka, Japan). The EtOAc layer was evaporated to dryness, and the residue was dissolved in methanol (MeOH). The supernatant, collected via filtration, was used for LC/MS analysis.

In the case of *S. nodosus*, colonies were harvested from the agar culture medium and extracted with ethanol (EtOH; Fujifilm Wako Pure Chemical Corp., Osaka, Japan). The extract was concentrated *in vacuo* to remove EtOH and then dissolved in MeOH; the supernatant, collected *via* filtration, was used for LC/MS analysis.

### Raman Spectroscopic Measurements

Two Raman spectrometers were employed herein. A custom-made Raman spectrometer, designed by HORIBA, Ltd., was used for the colony measurements, as depicted in the Supporting Information (Figure S1). The irradiation laser was set at 532 nm, with a power ranging from 0.5 to 5 mW. Four objective lenses (100×: 0.90-NA, 50×: 0.75-NA, 40×: 0.65-NA, and 10×: 0.25-NA) were used for the *E. coli* K12 measurements, and a 100× objective lens was employed for the actinomycete ones. The spectral resolution was 18.7 cm^–1^. For the *in situ* colony measurements, glass bottom dishes (D11140H, Matsunami Glass Ind., Ltd., Osaka, Japan) with caps were used as culture dishes. During the measurements, cover glasses (NEO Cover Glass 24 mm × 50 mm, Matsunami Glass Ind., Ltd., Osaka, Japan) were attached on top. Raman imaging measurements covered the entire colony, with a step size of 150–200 μm. The laser exposure time was set to 5 s for *E. coli* K12 (and only 2 s when using the 10× objective due to strong autofluorescence and the detection limit of the CCD detector), 1 s for *S. coelicolor* A3(2), and 2 s for *S. nodosus*.

The XploRA Plus Raman microscope (Horiba, Ltd., Kyoto, Japan) was used to collect reference spectra and for Raman cell imaging. The irradiation laser was set at 532 nm, with a power range of 0.4 to 10 mW, using a high-magnification objective lens (100×: 0.90-NA). The spectral resolution was 10.6 cm^–1^. For the Raman measurements to collect reference biomolecular Raman spectra, microbial mycelia were transferred from agar dishes to a quartz glass slide. The mycelium on the glass was gently washed with Milli-Q water to remove the medium and then dried at room temperature before being placed on the microscope stage for the Raman spectral measurements. *S. coelicolor* A3(2), *S. thermocarboxydus*, *Thermomonospora* sp., and *Saccharopolyspora* sp. were used to collect reference Raman spectra. Each strain was cultured on a WAP medium, and the Raman spectra were measured on culture days 2, 5, and 10. The mapping Raman measurements were conducted with a step size of 2 to 3 μm and a 10 s acquisition time. Under each condition, approximately 1000 Raman spectra were acquired. The mycelia derived Raman spectra of each strain were combined to one matrix, and analyzed by MCR-ALS. MCR resolved biomolecular Raman spectra were collected and utilized as reference spectra in the spectral analysis explained in the next section. For Raman cell imaging, *S. coelicolor* A3(2) was cultured under the same conditions mentioned above, but the step size was set to 0.36 μm for high-resolution Raman imaging.

### Raman Spectral Analysis

The recorded Raman spectra were divided by white light spectrum to calibrate the sensitivity derived from the detector. Cosmic ray was removed if existed. Wavenumber calibration was performed by fitting the peak positions of the Raman spectra of indene by fourth-order polynomial functions. And noise reduction was performed *via* singular value decomposition (SVD) by reconstructing the spectra by 20–30 SVD components before MCR-ALS analysis.^20,21^ The preprocessing was performed by IGOR Pro software (WaveMetrics, Inc., Lake Oswego, OR).

**Figure 1:**
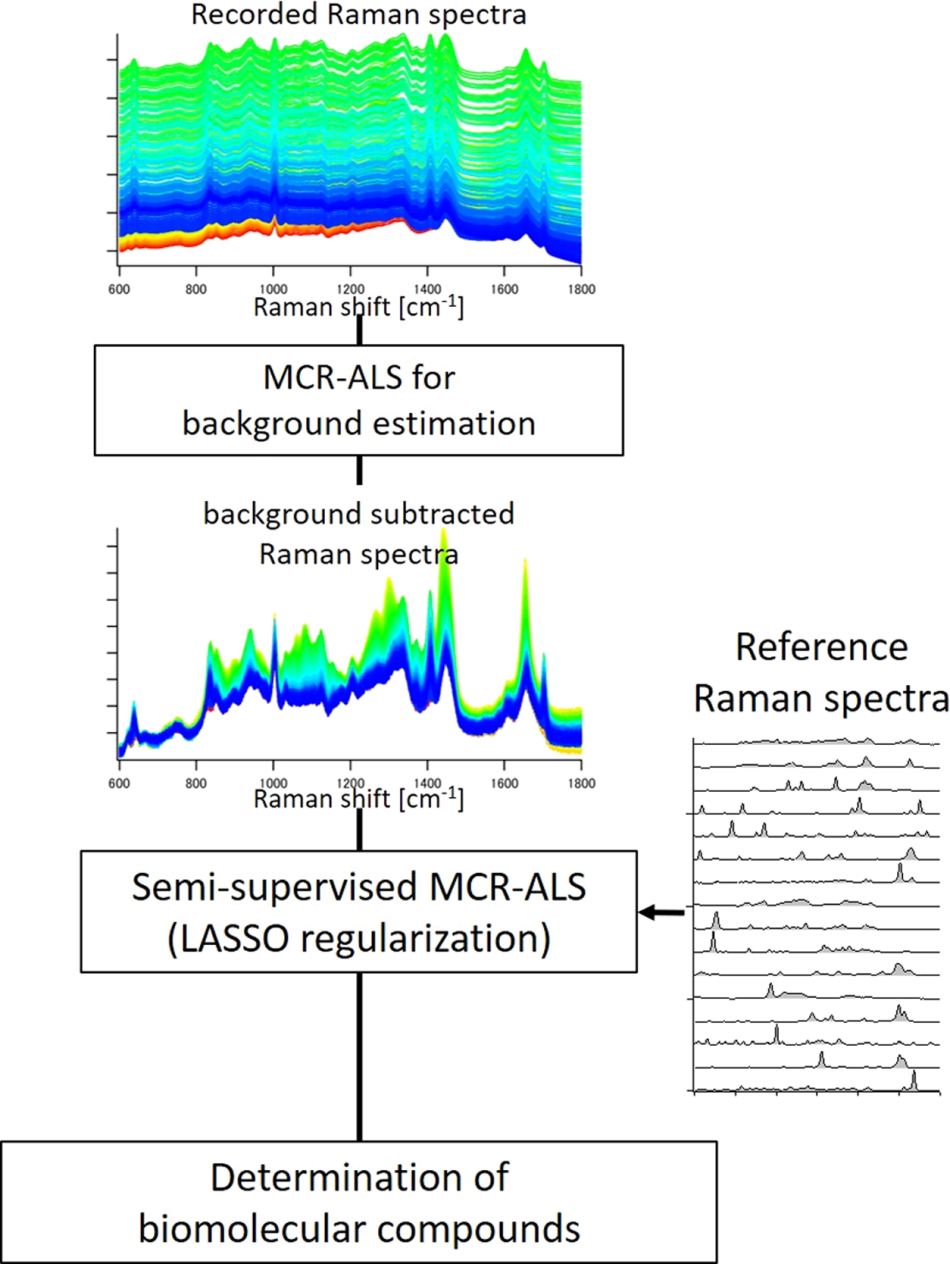
Flowchart of colony derived Raman spectral analysis including semisupervised MCR-ALS.

Spectral decomposition was performed using MCR-ALS calculations. The obtained Raman spectrum matrix A (rows for wavenumber, and columns for spectra)was decomposed into two matrices using the MCR-ALS method: ***A*** = ***WH*** + ***E***, where ***W*** refers to the MCR spectral components (rows for wavenumber, and columns for spectral components), ***H*** to the intensity profile corresponding to the spectral components(rows for intensity profile of components, and columns for concentration profile), and ***E*** to the residual.^20,22−24^ The Raman spectra of the colonies were analyzed by the following process (Figure 1). First, colony derived Raman spectra were preprocessed by the MCR-ALS to remove the autofluorescence background. In this process, components with broad spectral features without sharp band shapes were extracted as fluorescent components. Then, the contributions of the fluorescent spectral components were calculated based on the intensity information obtained from the **H** matrix, and subtracted from the original spectra to remove the fluorescent background. Subsequently, a semisupervised MCR-ALS analysis was performed for the secondary metabolite screening, in which reference spectra were utilized for initializing the MCR-ALS calculations. Here, the ALS optimization calculation allowed for flexible modification of the reference spectra within a range of 0.9 to 0.99 of cosine similarity with the initial spectra; correcting for variations in the reference spectra due to differences in machine and molecular existing environment, etc. In addition, sparse analysis with LASSO regularization was applied to accurately select the components that exist in the colony.^20,25^ Here, cross-validation was used in the optimization of hyperparameters. Furthermore, in the semisupervised MCR-ALS, optimization was performed for additional components not present in the reference spectra through random value initial estimate components. This was intended to extract spectra of unknown metabolites present in the colony. To confirm the validity of this analysis, artificially created Raman spectral data set were analyzed in Supporting material, and several biomolecules were successfully detected with accuracy (Figures S2–S14). The MCR-ALS calculations were performed using the Python SciPy library. The MCR resolved Raman images of the microbial colonies were smoothed *via* cubic polynomial interpolation with a 4 × 4 neighborhood value.

## Results and Discussion

### Optimization of Raman Measurements of Microbial Colonies

**Figure 2:**
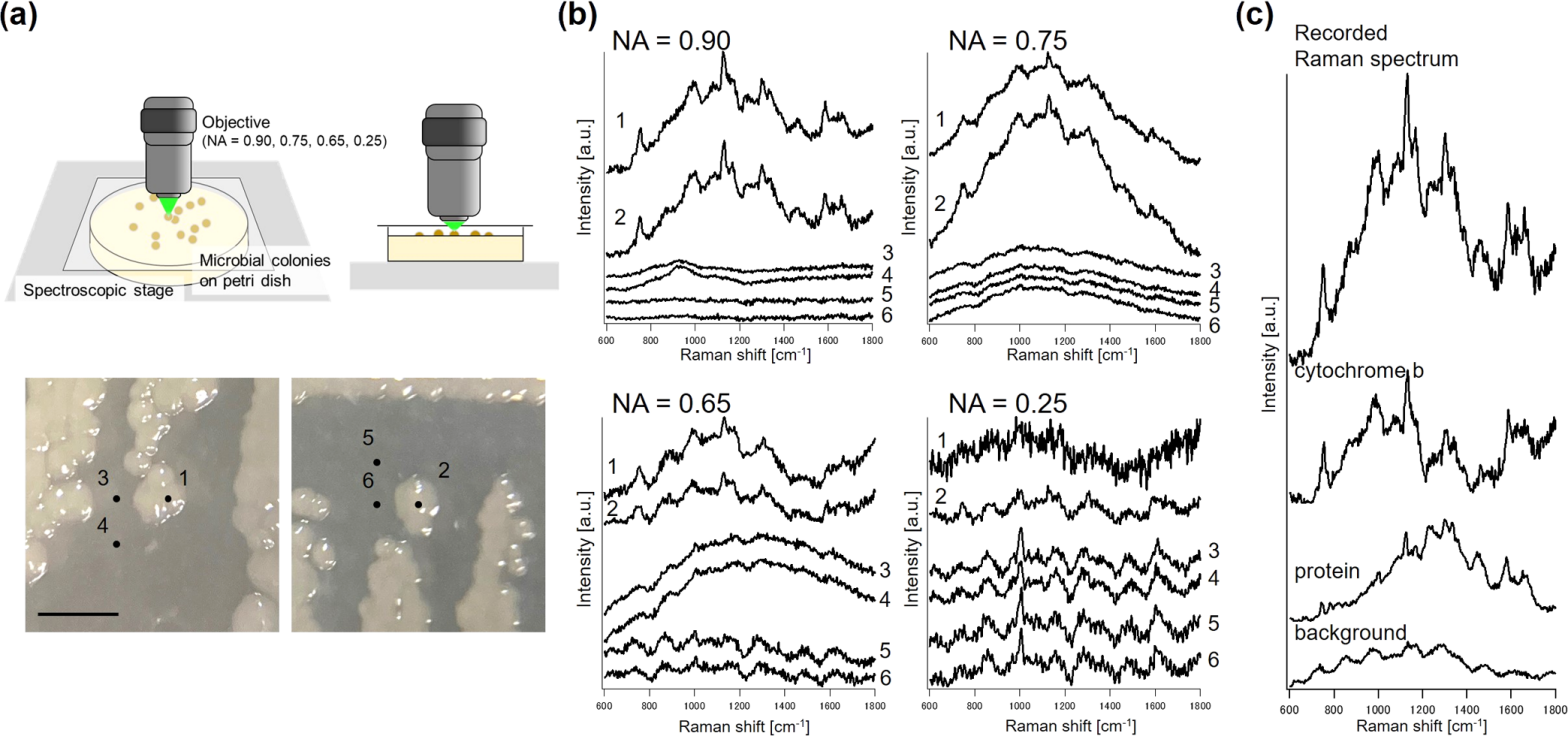
Colonies of *E. coli* K12 on agar plate and the obtained Raman spectra. (a) Images of the culture dish and stereo microscope images of *E. coli* K12. (b) Raman spectra obtained from each point (1 and 2, colony; 3, 4, 5, and 6, nonmicrobial area) using the corresponding objectives. (c) Recorded Raman spectra (NA = 0.90) and resolved biomolecular Raman spectra using the MCR-ALS. Scale bar = 2 mm.

The standard Petri dish is not suitable for *in situ* colony Raman spectroscopic measurements owing to its thickness, which disables the usage of high numerical aperture (NA) objectives, and plastic covers, which significantly interfere with Raman measurements. In this study, culture dishes suitable for colony Raman measurements were used; caps as thick as the cover glass were employed, and agar with culture medium was placed in the dish with the proper heights to enable the utilization of high-NA objectives. Furthermore, a Raman spectrometer suitable for the measurements was designed, with a transmission grating to ensure minimum loss of the Raman signals. Raman spectroscopic measurements of *E. coli* K12 colonies were conducted using various NA objectives to assess the requirements for the colony Raman measurements (Figure 2).

As shown in Figure 2a, a cover glass was placed immediately above the colony and irradiated with a laser. The background-subtracted Raman spectra reveal several bands indicative of biomolecules (Figure 2b). Notably, the bands around 750, 1130, 1304, and 1580 cm^–1^ are characteristic of Cytochrome b,^26^ while those at 1450 and 1660 cm^–1^, representing C–H bending and amide I vibrations, are associated with proteins.^27^ The signal-to-noise (S/N) ratio of the obtained Raman spectra was calculated *via* Gaussian fitting to these Raman bands and by using the standard deviation of the noise calculated from the linear fitting to the silent region (2000–2100 cm^–1^; Table 1). Generally, a positive correlation was observed between the calculated S/N ratio and the NA of the objective. The values derived from the Cytochrome *b* bands were approximately 2 to 3, even in the spectra obtained with low-NA objectives, while the values for proteins were lower. The stronger signal from Cytochrome b can be interpreted as arising from the resonance Raman effect.^26^ Protein bands were almost imperceptible in the spectra obtained with low-NA objectives (0.65 and 0.25).

**Table 1:** Optical Conditions and Calculated S/N Ratios of Raman Spectra Obtained from Two *E. coli* K12 Colonies

	spatial resolution [μm]		S/N ratio [-]			
NA	*XY*	*Z*	750 cm^–1^	1130 cm^–1^	1580 cm^–1^	1660 cm^–1^
0.90	1.2	6.4	5.28	8.15	6.12	4.65
0.75	2.3	7.8	3.49	4.16	1.90	2.27
0.65	2.8	8.6	2.44	3.25	1.90	1.92
0.25	11	∼240	2.13^a^	1.88^a^	1.52^a^	1.56^a^

a For *N* = 1 owing to the weak signal.

The spectra acquired with the 0.90-NA objective were decomposed *via* the MCR-ALS, where two biomolecular constituents (protein and Cytochrome b) were detected (Figure 2c). The result emphasizes that the Raman spectra of colonies tolerable for detailed biomolecular observations. Furthermore, the spectra acquired using low-NA objectives (0.65 and 0.25) exhibited specific bands at 1000 and 1610 cm^–1^, attributable to polystyrene, the material of the dish bottom (Figure S15). The NA of an objective influences the concentration of light irradiation on the focal plane. Low-NA objectives irradiate a larger area, leading to unintended signals from polystyrene. Typically, using high-NA objectives is desirable for acquiring high-S/N Raman spectra, though this is challenging considering their short focal lengths. Nevertheless, the culture apparatus devised herein successfully enabled the direct measurement of Raman spectra from colonies, even with high-NA objectives.

**Figure 3:**
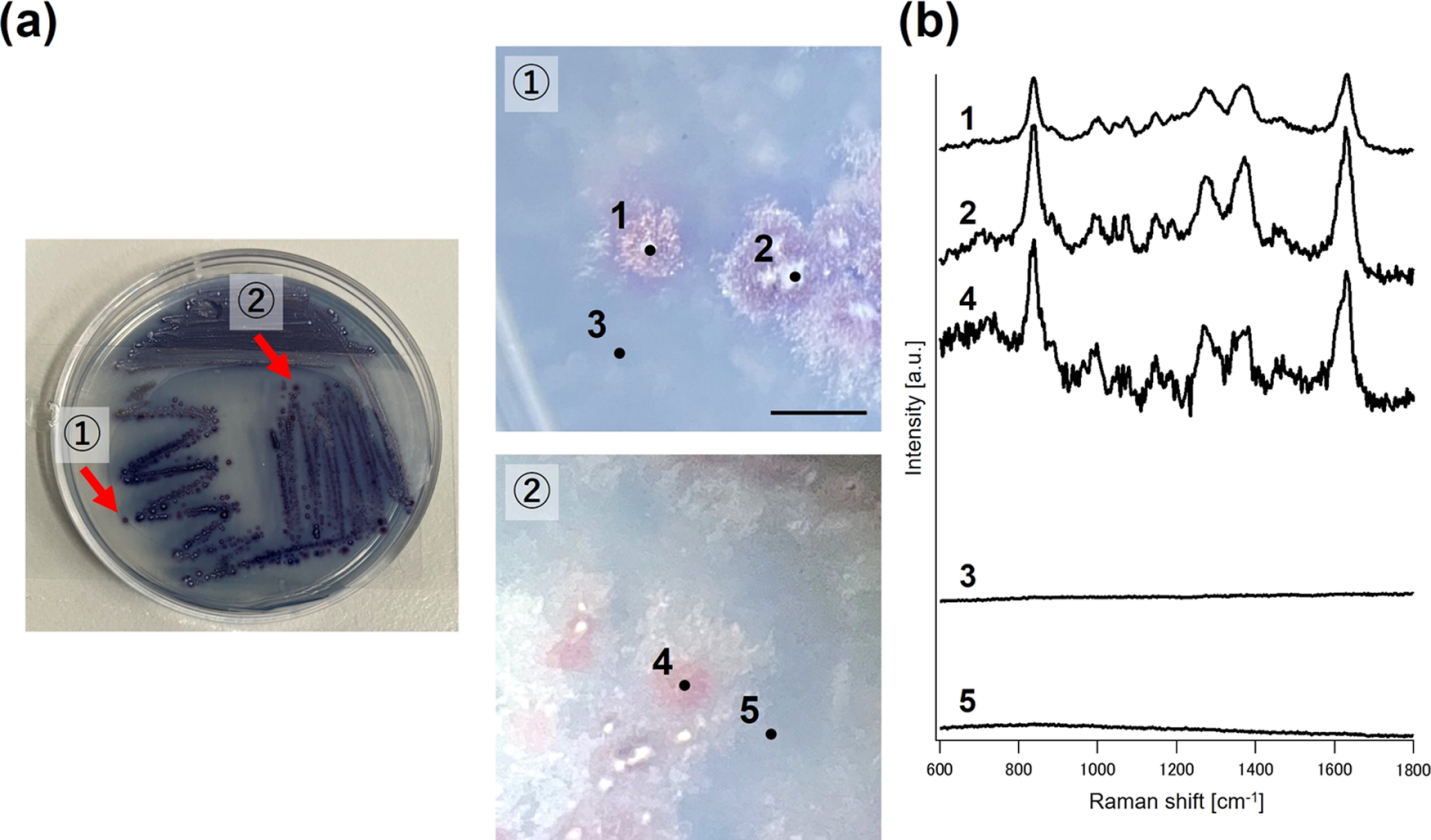
Microbial colonies of *S. coelicolor* A3(2) and measured Raman spectra. (a) Overall image of the culture dish and colonies of *S. coelicolor* A3(2), and stereoscopic images of the colonies. (b) Raman spectra obtained from each point (1, 2, and 4, on the colony; and 3 and 5, on the nonmicrobial area). Scale bar = 1 mm.

Subsequently, Raman measurements of *S. coelicolor* A3(2) colonies (cultured in a WAP medium for 9 days, Figure 3a) were performed. Due to the change in culture apparatus, we successfully obtained Raman spectra from actinomycetes (Figure 3b). *S. coelicolor* A3(2), known for producing the antibiotic undecylprodigiosin, exhibits characteristic Raman bands (1270, 1370, and 1630 cm^–1^) corresponding to this compound (Figure S16),^28^ as confirmed by LC/MS analysis (Figure S17). Thus, Raman measurements effectively analyzed not only *E. coli* colonies but also actinomycetes colonies that actively produce antibiotics.

### Preparation of Reference Biomolecular Raman Spectra from Actinomycetes

MCR-ALS can be used to estimate the biomolecular constituents of Raman spectra without prior information.^20,23^ However, Raman spectroscopic study of microbes sometimes suffers from strong autofluorescence and weak signals.^16,20^ In such cases, extracting clear Raman spectra of secondary metabolites is difficult, as was the case in this research. To obtain a robust MCR solution semiautomatically, the Raman spectra of known compounds were used in this study. Biomolecular Raman spectra were referenced during the MCR calculations. However, given the vast array of secondary metabolites produced by actinomycetes, preparing all the corresponding Raman spectra is impractical. Additionally, screening for novel compounds typically lacks prior information. Therefore, we herein proposed spectrum analysis utilizing semisupervised MCR analysis. Here, we aimed to extract information about known compounds using reference spectra while simultaneously performing spectrum extraction for compounds not included in the reference spectra as unknown components.

**Figure 4:**
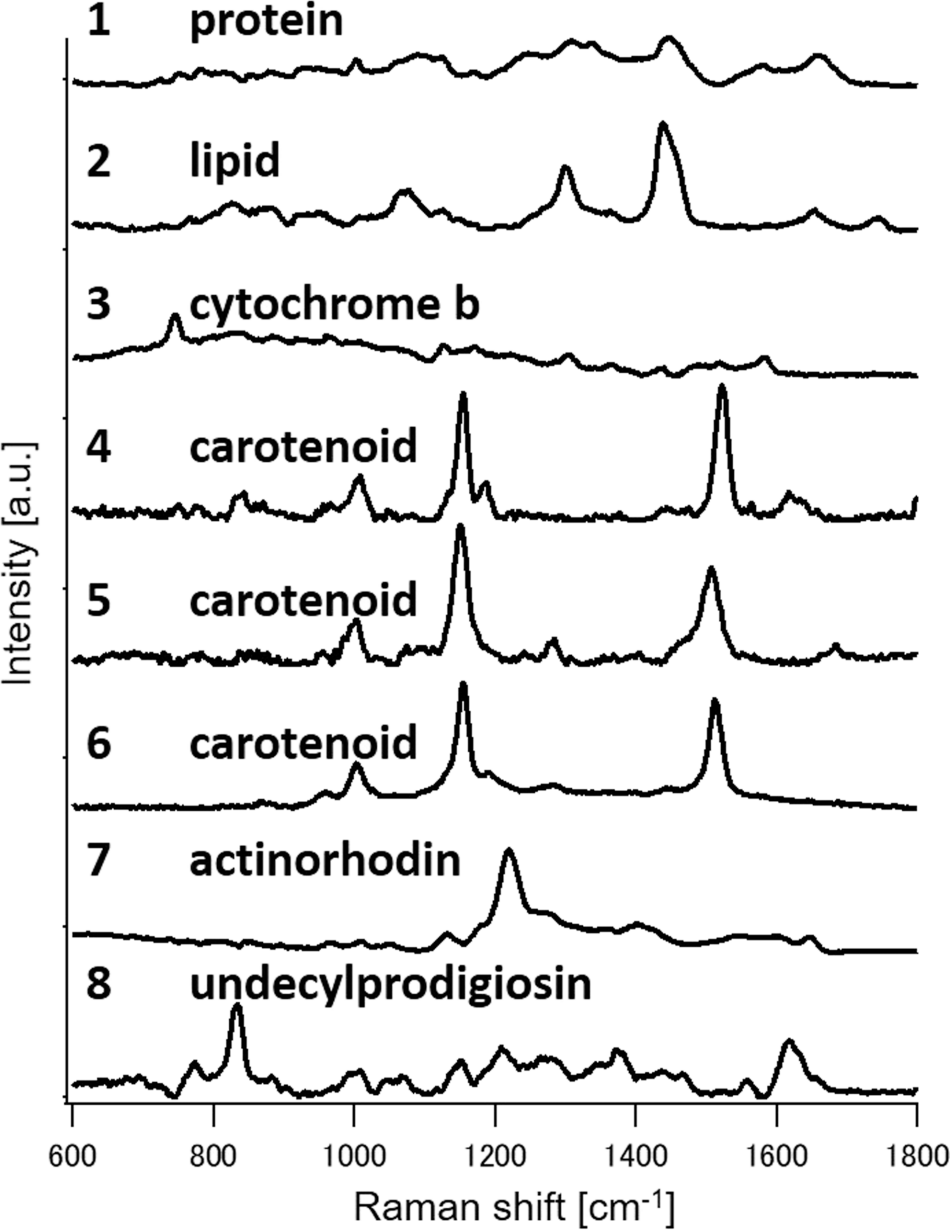
Biomolecular Raman spectra obtained from four actinomycetes Raman spectra resolved *via* MCR-ALS: (1) protein, (2) lipid, (3) Cytochrome b, (4–6) carotenoids, (7) actinorhodin, and (8) undecylprodigiosin.

To validate this approach, various biomolecular Raman spectra, including those of secondary metabolites, were prepared, and colony-derived Raman spectra were analyzed for secondary metabolite screening. The Raman spectra of four actinomycete strains’ mycelia, that were transferred to the slide glasses were measured, yielding MCR-resolved biomolecular spectra (Figure S18). From these, eight were selected and facilitated in MCR-ALS (Figure 4). Component 1 represents proteins, indicated by the bands at 1004 cm^–1^ (ring breathing mode of phenylalanine and tryptophan residues), 1270 cm^–1^ (amide III), 1449 cm^–1^ (C–H bending), and 1654 cm^–1^ (amide I).^27^ Component 2, corresponding to lipids, exhibits bands at 1080 cm^–1^ (C–C vibration), 1300 cm^–1^ (C–H2 twisting vibration), 1440 cm^–1^ (C–H2 deformation), 1650 cm^–1^ (C=C stretching vibration), and 1750 cm^–1^ (C=O vibration of the ester bond),^29,30^ suggesting a mixture of intracellular lipids with one or more C=C double bonds.^15^ Cytochrome b is identified as component 3, with bands at 746, 1126, 1304, 1335, and 1580 cm^–1^.^26^ Components 4 to 6 likely representing carotenoids, show bands at 1002–1007 cm^–1^ (C–CH3 deformation), 1155 cm^–1^ (C–C deformation), and 1520 cm^–1^ (C=C deformation),^31^ potentially corresponding to β-carotene or a mix of carotenoids, with slight peak shifts indicating different molecular aggregation states. Components 7 and 8, exclusive to *S. coelicolor* A3(2), correspond to the antibiotics actinorhodin and undecylprodigiosin, respectively.^32^ Component 7, representing actinorhodin, displays Raman bands at 1140, 1220, and 1650 cm^–1^,^33^ while component 8, assigned to undecylprodigiosin, is detailed in the previous section. The production of actinorhodin and undecylprodigiosin was confirmed between days 5 and 10 *via* LC/MS analysis (Figure S19).

### Time Course Imaging for Antibiotic Production by *S. coelicolor* A3(2)

The Raman imaging of antibiotic production by *S. coelicolor* A3(2) was performed. The Raman spectral data analyzed were collected from mycelia transferred from Petri dishes to the slide glass. The spatial distribution of various biomolecular components was analyzed *via* MCR-ALS by combining the all set of Raman images across time to one matrix (Figure S20). Components 1 to 5 represent proteins, actinorhodin, undecylprodigiosin, lipids, and carotenoids, respectively (the spectral assignments have been detailed earlier). Protein was homogeneously distributed within the cells. Both actinorhodin and undecylprodigiosin were detected exclusively on days 5 and 10, aligning with the LC/MS analysis presented earlier. Notably, the spatial distribution of these compounds varied, where undecylprodigiosin was found only within the cell, while actinorhodin was present both inside and slightly outside the cell. Scholars have previously observed actinorhodin within the cell, with some being exported outside *via* membrane vesicles.^33,34^ Raman imaging successfully captured the presence of secondary metabolites not only within the cells but also externally. Lipids were detected on days 2 and 5, localized within the cell on day 2 and evenly distributed by day 5. By day 10, lipids were undetectable. It is hypothesized that lipids contribute to actinorhodin production as fatty acids are a source of acetyl-CoA, a precursor for polyketide compounds.^35^ Carotenoids were detected minimally, localized within the cell. Thus, the Raman cell imaging effectively revealed secondary metabolite production by *S. coelicolor* A3(2) and its time-course changes.

### Analysis of *S. coelicolor* A3(2) Colony for Secondary Metabolite Production

**Figure 5:**
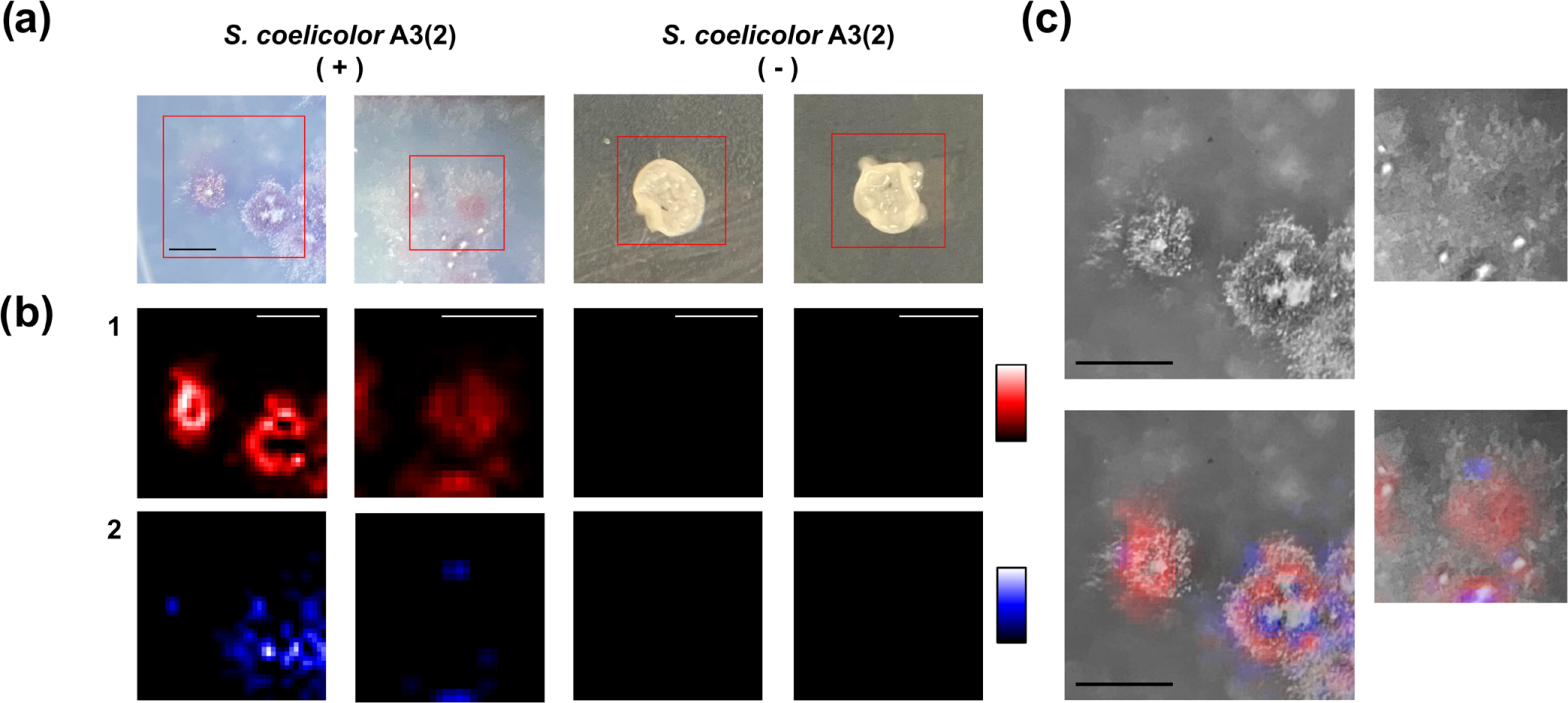
Raman imaging analysis of *S. coelicolor* A3(2) colonies on agar dishes. (a) Stereoscopic images of *S. coelicolor* A3(2) colonies under actinorhodin- and undecylprodigiosin-producing conditions (+) and nonproducing conditions (−). (b) Raman images of (1) undecylprodigiosin and (2) actinorhodin. (c) Overlay of Raman images on black-and-white stereoscopic colony images. Scale bar = 1 mm.

In this study, the utilization of semisupervised MCR analysis with reference spectra was aimed at visualizing the production and distribution patterns of known compounds within colonies. The secondary metabolite production in *S. coelicolor* A3(2) colonies (Figure 5a) was analyzed using semisupervised MCR (Figure S21a displays the recorded Raman spectra). Under conditions favoring actinorhodin and undecylprodigiosin production, both compounds were detected in the colonies. In contrast, under nonproducing conditions, the shape of the colonies changed and neither compound was detected (Figure 5b), aligning with the LC/MS analytical results (Figure S19). This analysis effectively distinguished the production of the two antibiotics using prior information (see Figure S22 for details of the MCR results). Additionally, the Raman measurements of *S. coelicolor* A3(2) colonies were notably efficient, averaging around 1 min (∼50 spectra measured to cover the entire single colony). This period is significantly shorter than that required for traditional metabolome analysis. However, in contrast to the cell measurements, biomolecular compounds other than these two were not detected. The strong autofluorescence emitted during the measurements is likely the main cause of this discrepancy. Compared to the cell Raman measurements, *in situ* colony measurements are significantly affected by the medium used for the culture, which is usually removed during sample preprocessing. This strong autofluorescence often obscures the presence of minor biomolecular components in Raman spectra, such as proteins, lipids, and carotenoids. Addressing these challenges is crucial for future high-throughput, high-sensitivity screening processes.

In addition to detecting these secondary metabolites, Raman spectroscopic imaging provided valuable biological insights into *Streptomyces* differentiation and secondary metabolism under solid culture conditions. *Streptomyces* species form mycelia extending to the agar medium and the air, called substrate and aerial mycelia, respectively.^16,36^ Though the production of secondary metabolites has been related to mycelial differentiation, the mechanisms have not been exhaustively studied. In this case, the top white part of the colonies is regarded as the aerial mycelium, whereas the purple-colored area constitutes the substrate mycelium. The distribution of the two compounds was different, as illustrated in Figure 5b,5c. While undecylprodigiosin aligned with the colony shape, actinorhodin was more widely distributed, with variations in production among colonies. Actinorhodin was particularly prominent at the edge of the aerial mycelium, where mycelial differentiation had progressed significantly. Studies have shown that *Streptomyces* secondary metabolism occurs in both the aerial and substrate mycelium.^16,36^ It has been reported that *S. coelicolor* A3(2) secretes aqueous droplets containing actinorhodin onto the colony surface postsporulation.^32,37^ The imaging herein may have captured actinorhodin accumulation within cells prior to secretion. Unlike undecylprodigiosin, actinorhodin was observed not only on the colonies but also around the periphery, consistent with studies demonstrating its secretion outside the mycelium.^33,34^ This *in situ* observation suggests that this screening method can detect extracellular compounds as well as intracellular ones.

### Exploring Secondary Metabolite Screening with *S. nodosus* Colonies

**Figure 6:**
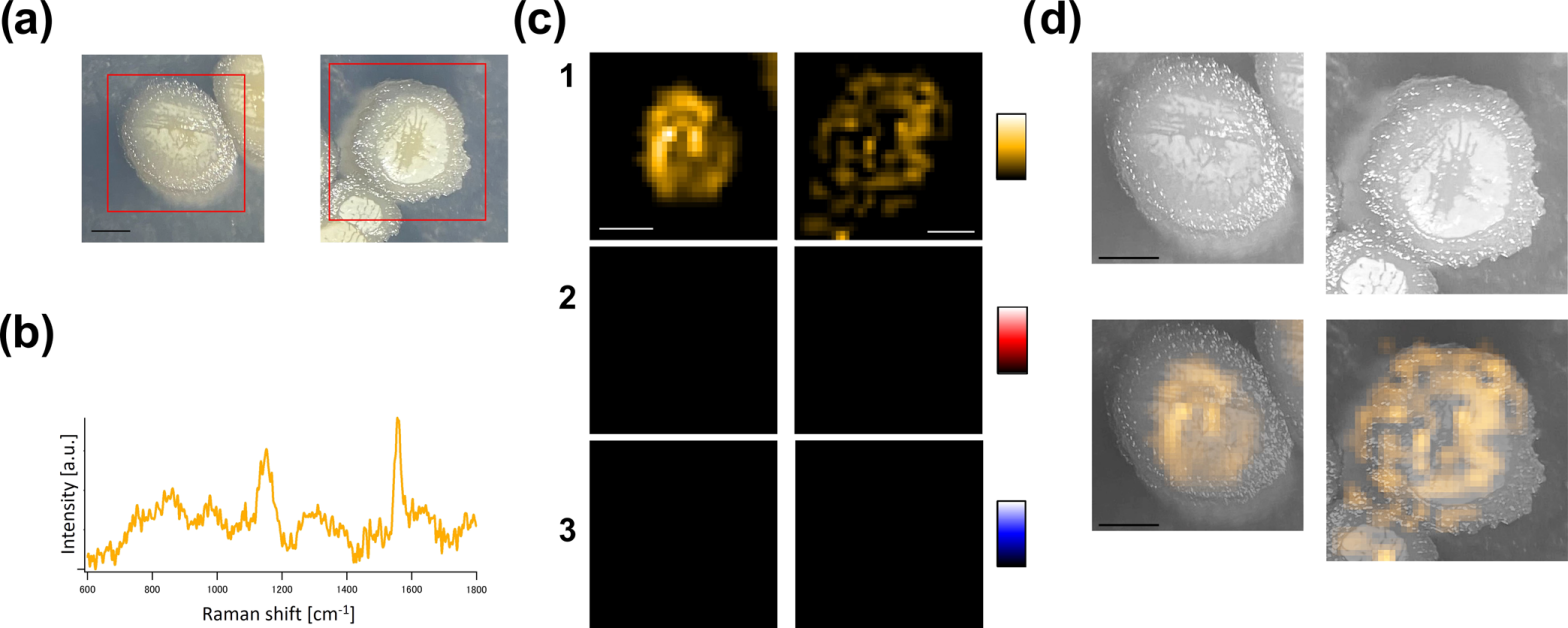
Raman imaging analysis of *S. nodosus* colonies on agar dishes. (a) Stereoscopic images of *S. nodosus* colonies under AmB-producing conditions. (b) Raman spectrum of AmB resolved using the MCR-ALS method. (c) Raman images of (1) AmB, (2) undecylprodigiosin, and (3) actinorhodin. (d) Overlay of Raman images on black-and-white stereoscopic colony images. Scale bar = 1 mm.

In semisupervised MCR, it also becomes feasible to extract spectra absent in the reference spectra through ALS optimization. This capability is expected to enable the exploratory detection of secondary metabolites. Using *S. nodosus* colonies as a model in this study, we verified the feasibility of extracting spectral components absent in the reference spectra. *S. nodosus* is known to produce AmB, which is recognized for its high antitumor activity and role in preventing fungal infections.^38^ Colonies cultivated under conditions conducive to AmB production (Figure 6a) were recorded, and subjected to semisupervised MCR analysis using the reference spectra, as mentioned above (see Figure S21b for the recorded Raman spectra). Initially, the intensity contributions of undecylprodigiosin and actinorhodin, included in the reference spectra, were found to be below the noise level, confirming their absence (see Figure S23 for the detailed MCR result and Figure S24 for the LC/MS results). On the other hand, an additional spectral component, as shown in Figure 6b, was extracted. The extraction of such an additional component may suggest the possibility of a new secondary metabolite not included in the reference data. It is possible to estimate the molecular structure based on the vibrational modes from the Raman band positions, and the compound can be identified by comparing the extracted spectra with the standard spectra of candidate compounds. In this case, peaks were detected at 1150 and 1550 cm^–1^, corresponding to polyene-derived C–C and C=C stretching, respectively. Comparing the spectrum with the authentic AmB spectrum,^39^ we confirmed that AmB was indeed produced by the *S. nodosus* colonies cultured under the specified conditions. The production of AmB was further validated through LC/MS analysis (Figure S24). This underscores the potential applicability of this technique toward discovering new compounds at the colony level. While conventional LC/MS and NMR analyses are still required for comprehensively determining the chemical structure of newly discovered secondary metabolites, the approach utilizing Raman spectroscopy can be useful in easily and nondestructively selecting prominent candidate target strains during screening.

The production of AmB exhibited heterogeneity across each *S. nodosus* colony, similar to the case for *S. coelicolor* A3(2) (Figure 6c,d). The central part of the colonies is regarded as the aerial mycelium, while the peripheral part involves the substrate mycelium.^36^ In the left colony Raman image of Figure 6c, strong AmB production was evident in the aerial mycelium but unobservable in the substrate mycelium. This could be due to the absence of AmB production in the substrate mycelium. On the other hand, in the right colony image of the Figure 6c, AmB production was observed in both areas, albeit heterogeneously. This outcome may reflect the complex mechanisms of mycelial differentiation and secondary metabolism during colony formation. *In situ* Raman spectroscopic colony observations hold the potential for not only detecting secondary metabolites but also providing new biological insights into *Streptomyces* differentiation and secondary metabolism.

## Conclusions

In this study, we conducted the direct measurement of microbial colonies on agar dishes, a previously unachievable task, by preparing the appropriate culture apparatus. We observed that the S/N ratio of the Raman spectra obtained from *E. coli* varied with the NA of the objectives, underscoring the importance of using high-NA objectives for Raman measurements of microbial colonies. Additionally, the Raman measurement of *S. coelicolor* A3(2) colonies was notably efficient, averaging around 1 min; this is significantly shorter than the period required for traditional metabolome analysis. With further improvements in the measurement conditions, we anticipate that the exhaustive measurement of colonies isolated from the environment will become feasible.

The spectral analysis for secondary metabolite screening yielded promising results. The production of compounds with known spectral profiles was accurately identified *via* semisupervised MCR analysis. Notably, even without prior spectral information, we successfully detected the production of AmB by *S. nodosus* by extracting specific Raman spectra, showcasing the method’s potential for discovering new secondary metabolites. Furthermore, from the Raman spectroscopic study of the colonies, we observed heterogeneity in antibiotic production in the colonies, indicating the applicability of this method toward not only detecting these compounds but also investigating the biological aspects of colony phenotypes. This technological advancement heralds a new era in the rapid discovery of novel compounds, potentially revolutionizing the field.

## Supporting Information Available

Spectrometer setup, demonstration of semisupervised MCR-ALS, LC/MS spectra, and supplemental Raman spectral information (PDF)
