# Supplementary material for: In Situ Raman Hyperspectral Analysis of Microbial Colonies for Secondary Metabolites Screening
Source: Anal Chem. 2024 Aug 31;96(37):14909–17. doi: 10.1021/acs.analchem.4c02906 (PMC11411491; doi:10.1021/acs.analchem.4c02906)
Supplement: Supplementary file 1 — ac4c02906_si_001.pdf [file ac4c02906_si_001.pdf]

Supporting Information

***In Situ* Raman Hyperspectral Analysis of Microbial Colonies for Secondary Metabolites Screening**

Shunnosuke Suwa,[†,‡] Masahiro Ando,[*,§] Takuji Nakashima,[§] Shumpei Horii,[†] Toyoaki Anai,[‖] Haruko Takeyama,[*,†,‡,§,⊥,#]

[†] Department of Advanced Science Engineering, Graduate School of Advanced Science and Engineering, Waseda University, 3-4-1 Okubo, Shinjuku-Ku, Tokyo 169-8555, Japan

[‡] Computational Bio Big-Data Open Innovation Laboratory (CBBD-OIL), National Institute of Advanced Industrial Science and Technology, 3-4-1 Okubo, Shinjuku-Ku, Tokyo 169-8555, Japan

[§] Research Organization for Nano and Life Innovation, Waseda University, 513 Wasedatsurumaki-Cho, Shinjuku-Ku, Tokyo 162-0041, Japan

[‖] Faculty of Agriculture, Kyushu University, 744 Motooka, Nishi-ku, Fukuoka, Fukuoka, 819-0395 Japan

[⊥] Institute for Advanced Research of Biosystem Dynamics, Graduate School of Advanced Science and Engineering, Waseda Research Institute for Science and Engineering, Waseda University, 3-4-1 Okubo, Shinjuku-Ku, Tokyo 169-8555, Japan

[#] Department of Life Science and Medical Bioscience, Graduate School of Advanced Science and Engineering, Waseda University, 2-2 Wakamatsu-Cho, Shinjuku-Ku, Tokyo 162-8480, Japan

*Corresponding author:
Masahiro Ando (mando@aoni.waseda.jp)
Haruko Takeyama (haruko-takeyama@waseda.jp)

Table of Contents

Figure S1 .... S3

Proof of concept for semi-supervised MCR-ALS (Figure S2 ~ S14)

Figure S2 .... S4
Figure S3 .... S5
Figure S4 .... S6
Figure S5 .... S6
Figure S6 .... S7
Figure S7 .... S8
Figure S8 .... S8
Figure S9 .... S9
Figure S10 .... S10
Figure S11 .... S10
Figure S12 .... S11
Figure S13 .... S12
Figure S14 .... S12

Figure S15 .... S13
Figure S16 .... S13
Figure S17 .... S13
Figure S18 .... S14
Figure S19 .... S15
Figure S20 .... S16
Figure S21 .... S17
Figure S22 .... S18
Figure S23 .... S19
Figure S24 .... S20

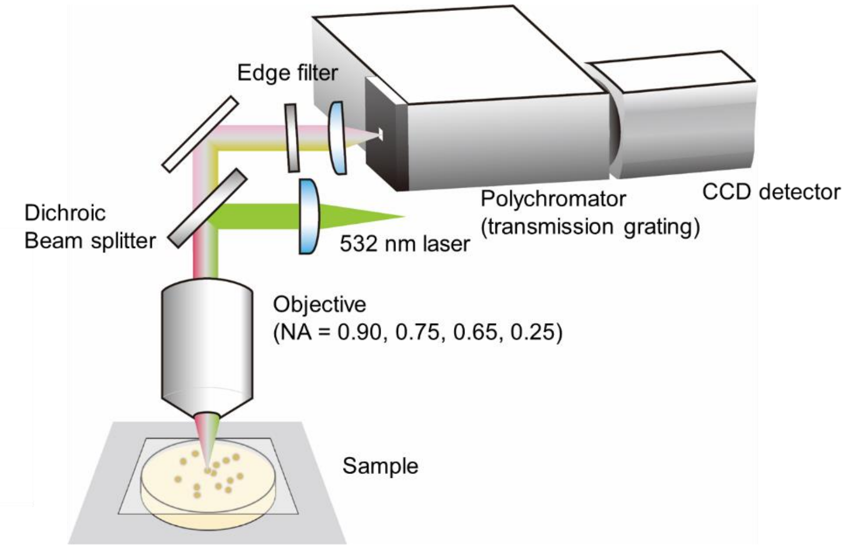

Figure S1. The schematic diagram of custom-made Raman spectrometer

Proof of concept for semi-supervised MCR-ALS by using artificial Raman spectral dataset

Collection of the standard Raman spectra

The concept of semi-supervised MCR-ALS was demonstrated by analyzing artificial Raman spectral dataset that mixed standard Raman spectra of biomolecular compounds, artificially created background, and random noise. The standard compounds used for the experiment are the following, albumin (Fujifilm Wako Pure Chemical Co., Tokyo, Japan), oleic acid (Tokyo Chemical Industry Co., Ltd., Tokyo, Japan), palmitic acid(Fujifilm Wako Pure Chemical Co., Tokyo, Japan), sodium pyruvate (Fujifilm Wako Pure Chemical Co., Tokyo, Japan), citric acid (Fujifilm Wako Pure Chemical Co., Tokyo, Japan), L(+)-ascorbic acid (Fujifilm Wako Pure Chemical Co., Tokyo, Japan), ergosterol (Tokyo Chemical Industry Co., Ltd., Tokyo, Japan), soluble starch (Fujifilm Wako Pure Chemical Co., Tokyo, Japan), lincomycin (Tokyo Chemical Industry Co., Ltd., Tokyo, Japan), pyridoxine(Kanto Chemical Co., Inc., Tokyo, Japan), sparsomycin (Cayman Chemical Company, Inc., Michigan, USA), streptomycin (Fujifilm Wako Pure Chemical Co., Tokyo, Japan), trans-feluric acid (Tokyo Chemical Industry Co., Ltd., Tokyo, Japan), benzylpenicillin potassium (Fujifilm Wako Pure Chemical Co., Tokyo, Japan), trans-o-coumaric acid (Tokyo Chemical Industry Co., Ltd., Tokyo, Japan), and avermectinB1a (Fujifilm Wako Pure Chemical Co., Tokyo, Japan). All of the compounds except for oleic acid were measured as powders (Figure S2).

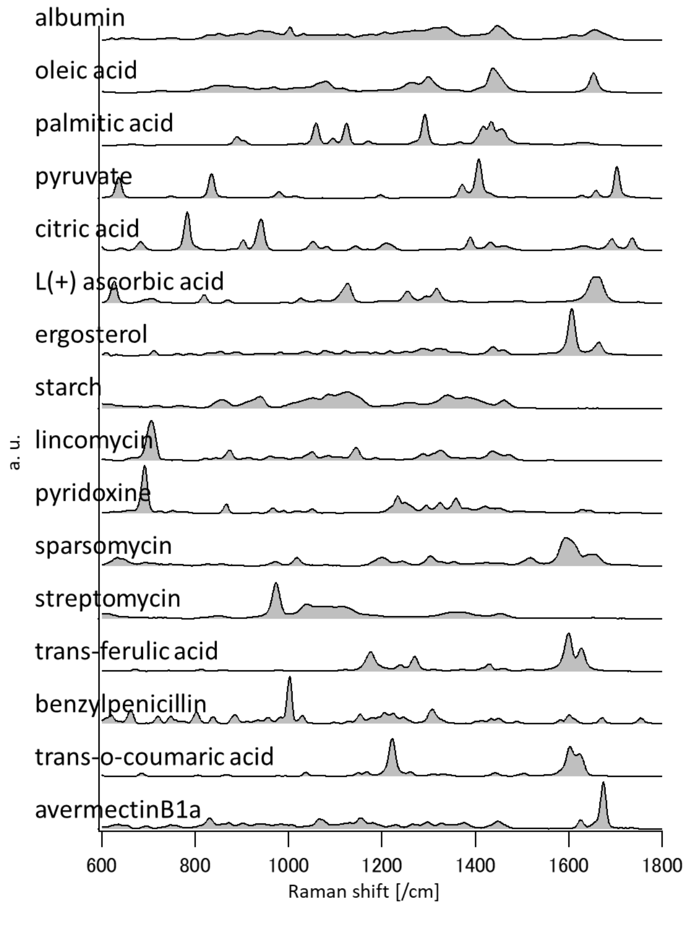

Figure S2. Recorded standard Raman spectra used for demonstrating semi-supervised MCR-ALS

1. Semi-supervised MCR-ALS: six compounds Raman spectral dataset

To demonstrate the sparse spectral decomposition of the dataset with the small numbers of components by a large number of reference spectra, the spectral dataset with six biomolecular components were prepared. These Raman spectral components were mixed with the following intensity profile (25 x 25 grid) with artificially created background components and random noise component (Figure S3a).

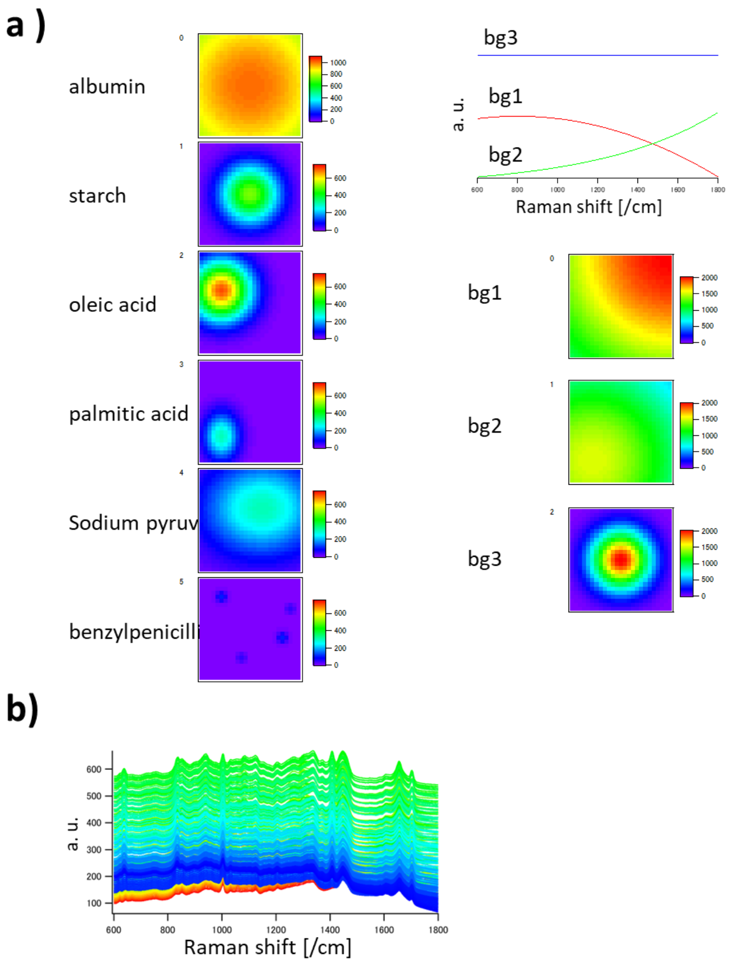

Figure S3. Raman intensity profiles set for artificial Raman spectral dataset. a) The intensity profile of each biomolecular and background component. b) Prepared Raman spectral dataset.

For the tailored Raman spectral dataset (Figure S3b), background subtraction was performed by MCR-ALS: **A** = **WH** + **E**. In this process, components with broad spectral features without sharp band shapes were extracted as background components. Then, the contribution of the background spectral components was calculated based on the intensity information obtained from the **H** matrix, and subtracted from the original spectra to remove the background. The resulting Raman spectra indicated the background mostly removed, while preserving biomolecular Raman spectral feature (Figure S4).

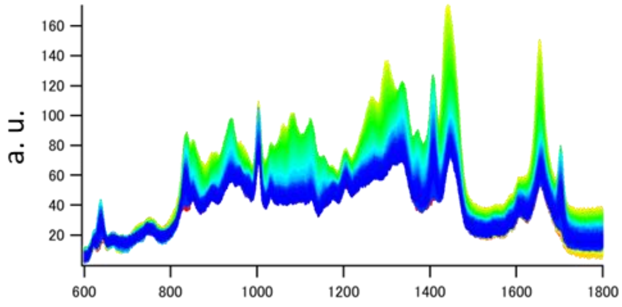

Figure S4. Background subtracted Raman spectral dataset

For the background subtracted dataset, semi-supervised MCR-ALS analysis was performed by using reference spectra of previously mentioned compounds except for benzylpenicillin and pyruvate (Figure S2). Benzylpenicillin and pyruvate were regarded as unknown compounds to mimic the discovery of new biomolecules being detected with no reference spectra in the calculation. In the ALS calculation, spectral variation was allowed for the reference spectra in the range of 0.999 in cosine similarity.

In MCR-ALS, it is generally necessary to select a matrix rank to estimate the number of components. However, in the semi-supervised MCR used in this study, it is assumed that a large number of spectral components are utilized as reference spectra. Therefore, instead of estimating the rank in the matrix decomposition, we apply a LASSO regularization term to obtain a sparse solution.

$$\underset{W\geq 0, H\geq 0}{\arg\min}(\|\boldsymbol{A}-\boldsymbol{WH}\|_2 + \alpha_{L1H}\|\boldsymbol{H}\|_1)$$

The hyperparameter α_{L1} of the LASSO regularization term are optimized by cross-validation, which evaluates the L2 norm of the residuals of the MCR result matrix. The data set was subjected to 5-fold cross-validation to estimate the α_{L1H} values (Figure S5). After cross-validation, appropriate α_{L1H} values were estimated to be around 1e-06 to 1e-05.

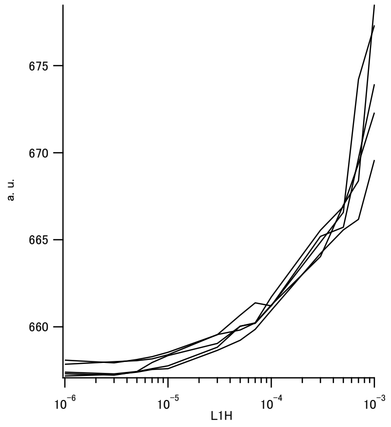

Figure S5. Cross-validation for searching α_{L1H} for semi-supervised MCR-ALS.

Here, various values of α_{L1H} (0, 1e-05, 8e-03) was applied for semi-supervised MCR-ALS to confirm the accuracy (Figure S6), by using reference spectra of albumin, oleic acid, palmitic acid, citric acid, L(+)-ascorbic acid, ergosterol,

starch, sparsomycin, streptomycin, lincomycin, avermectinB1a, pyridoxine, trans-ferulic acid, and trans-o-coumaric acid. The result demonstrated setting proper L1H value effective for detecting compounds accurately. In the case of α_{L1H} = 1e05, the components were detected with high accuracy and sparseness, including unknown compounds benzylpenicillin and pyruvate as well (Figure S6 and S7). As shown in Figure S8, the proper α_{L1H} value allowed accurate extraction of Raman spectra of benzylpenicillin and pyruvate. The comparison of the extracted spectra and standard spectra showed the possibility of exploring the unknown compounds with the pure Raman spectral information.

In the case of α_{L1H} = 0, while existing compounds were generally accurately detected, several kinds of unincluded compounds were detected incorrectly, such as citric acid, streptomycin or avermectinB1a (Figure S6). In the case of α_{L1H} = 8e-03, many of the components were not detected accurately, such as albumin, oleic acid, palmitic acid, and starch. Moreover, Raman spectra of benzylpenicillin and pyruvate included as unknown compounds were not extracted through calculation.

Consequently, the analysis allowed sparse biomolecular detection in the spectral dataset using large numbers of reference Raman spectra, succeeding in detecting unknown compounds simultaneously.

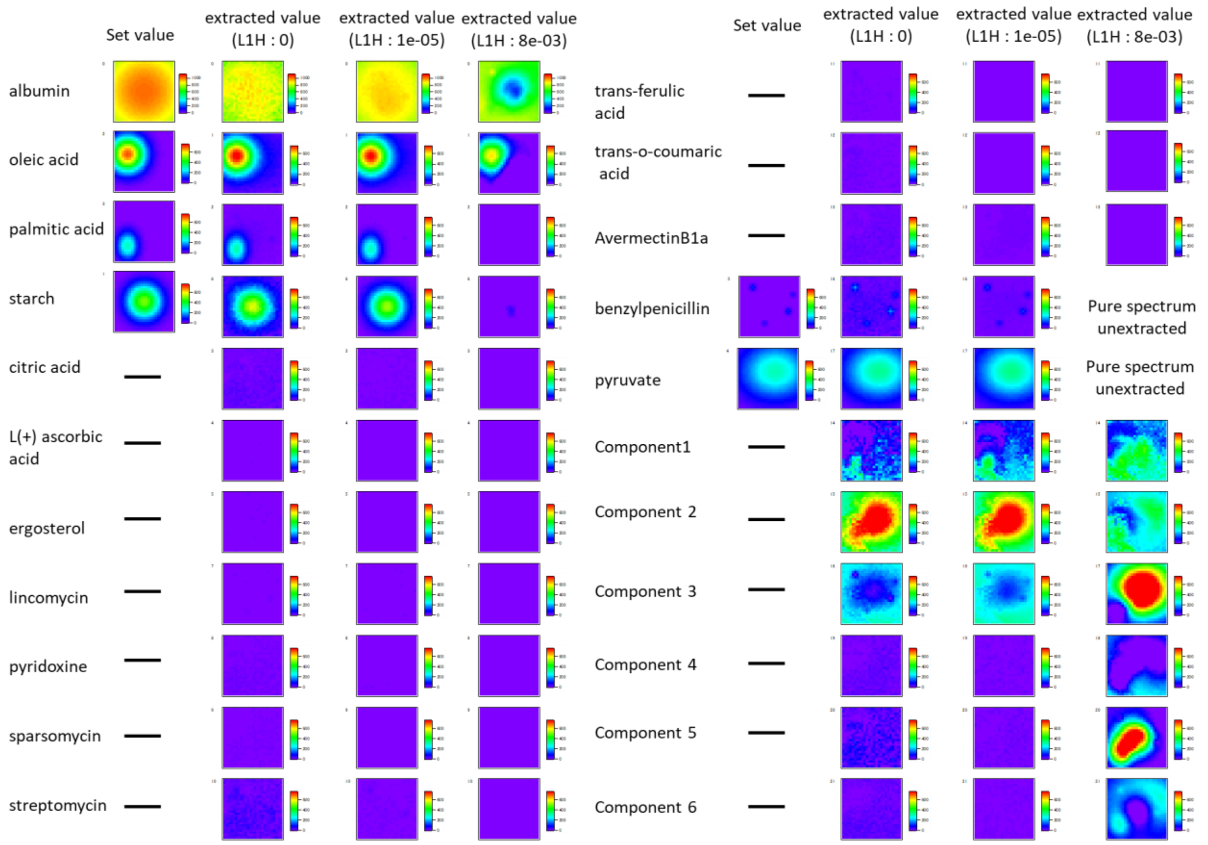

Figure S6. Comparison of Raman spectral intensity profile between set and resolved via MCR-ALS.
" – " indicates the component was not used for preparing the dataset.

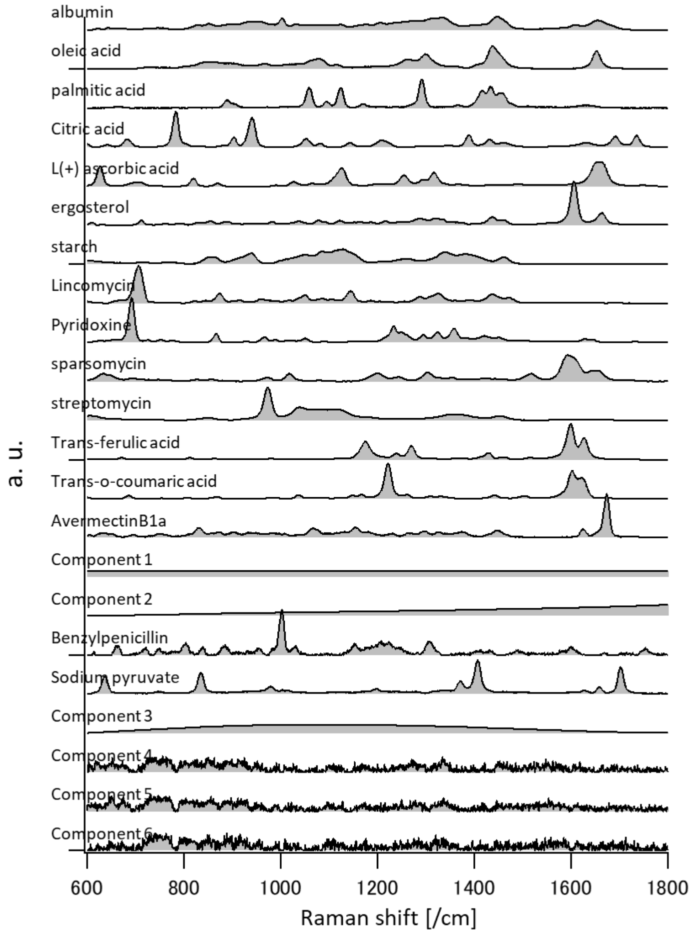

Figure S7. semi-supervised MCR resolved spectra for six components Raman spectral dataset with L1H of 1e-05. Benzylpenicillin, sodium pyruvate and Component 1 – 6 were detected during calculation without reference spectra.

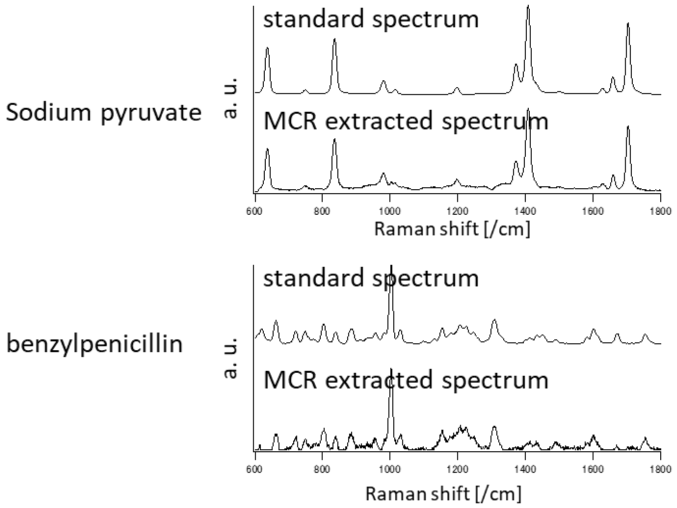

Figure S8. Raman spectra of sodium pyruvate and benzylpenicillin obtained by semi-supervised MCR-ALS (L1H: 1e-05), without reference Raman spectral information.

2. Semi-supervised MCR-ALS: 14 compounds Raman spectral dataset

To confirm the validity of this analysis for Raman spectra with a larger mixture of components, the spectral dataset with 14 biomolecular compounds were prepared and used for the demonstration. The 14 spectral components and artificial background were mixed with the following profile (Figure S9a). As in the previous demonstration, random noise component was also added. The tailored Raman spectra had very strong background components (Figure S9b)

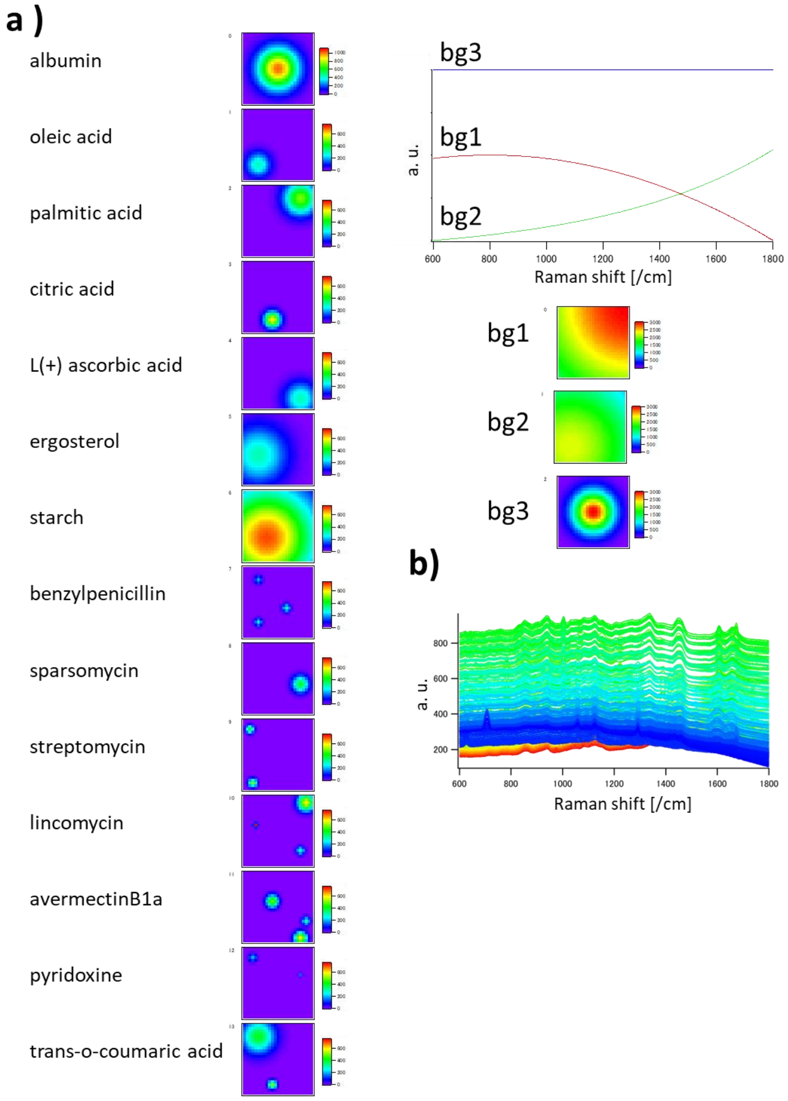

Figure S9. Raman intensity profiles set for artificial Raman spectral dataset. a) The intensity profile set for each biomolecular and background component. b) Prepared Raman spectral dataset.

Background subtraction was performed, using the same technique as int the previous demonstration. The resultant spectral dataset indicates the background spectra mostly removed, preserving Raman spectral profile (Figure S10b).

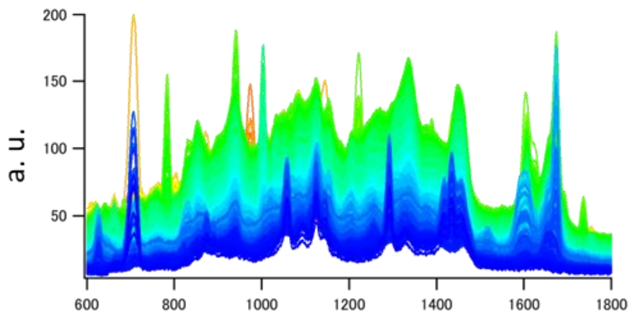

Figure S10. Background subtracted Raman spectral dataset

Semi-supervised MCR-ALS was then performed for the background-subtracted dataset, by using reference spectra of albumin, oleic acid, palmitic acid, citric acid, L(+)-ascorbic acid, ergosterol, starch, benzylpenicillin, sparsomycin, streptomycin, lincomycin, avermectinB1a, and pyruvate. (Figure S1). Pyridoxine and trans-o-coumaric acid were regarded as unknown compounds, to be detected with no reference spectra in the calculation, for the demonstration of the discovery of the new compounds. As in the previous demonstration, cross-validation was performed to estimate the proper hyperparameter α_{L1H}. The result indicated that the residuals drastically increased when α_{L1H} exceeds 1e-04. (Figure S11).

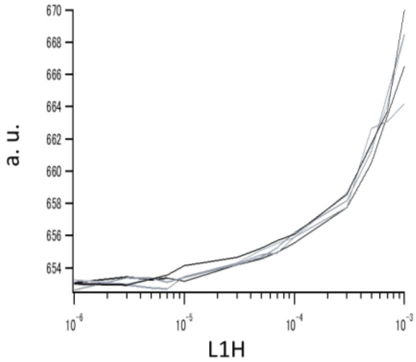

Figure S11. Cross-validation for L1H. The horizontal axis indicates L1H and the vertical axis indicates L2 norm of the residual

Here, semi-supervised MCR was performed with various α_{L1H} values (0, 4e-05, 9e-03). The results clearly showed that adjusting α_{L1H} allows us to evaluate biomolecular production with high accuracy. The spectral dataset was well decomposed with the α_{L1H} values at 4e-05 (Figure S12). The spectra extracted through the ALS calculation also included some additional components such as pyridoxine, trans-o-coumaric acid and others, that were assumed to be unknown component (Figure S13). Newly extracted pyridoxine and trans-o-coumaric acid spectra highly preserved Raman spectral feature of them (Figure S14).

Lower α_{L1H} value caused large errors in several components such as citric acid, benzylpenicillin and sparsomycin (Figure S12). On the other hand, larger α_{L1H} value caused crucial misfitting for albumin, L(+)-ascorbic acid, ergosterol, starch.

The results show that the analysis can be applied to Raman spectral data sets consisting of many components,

including unknown components, by applying a reference spectrum and LASSO regularized ALS optimization.

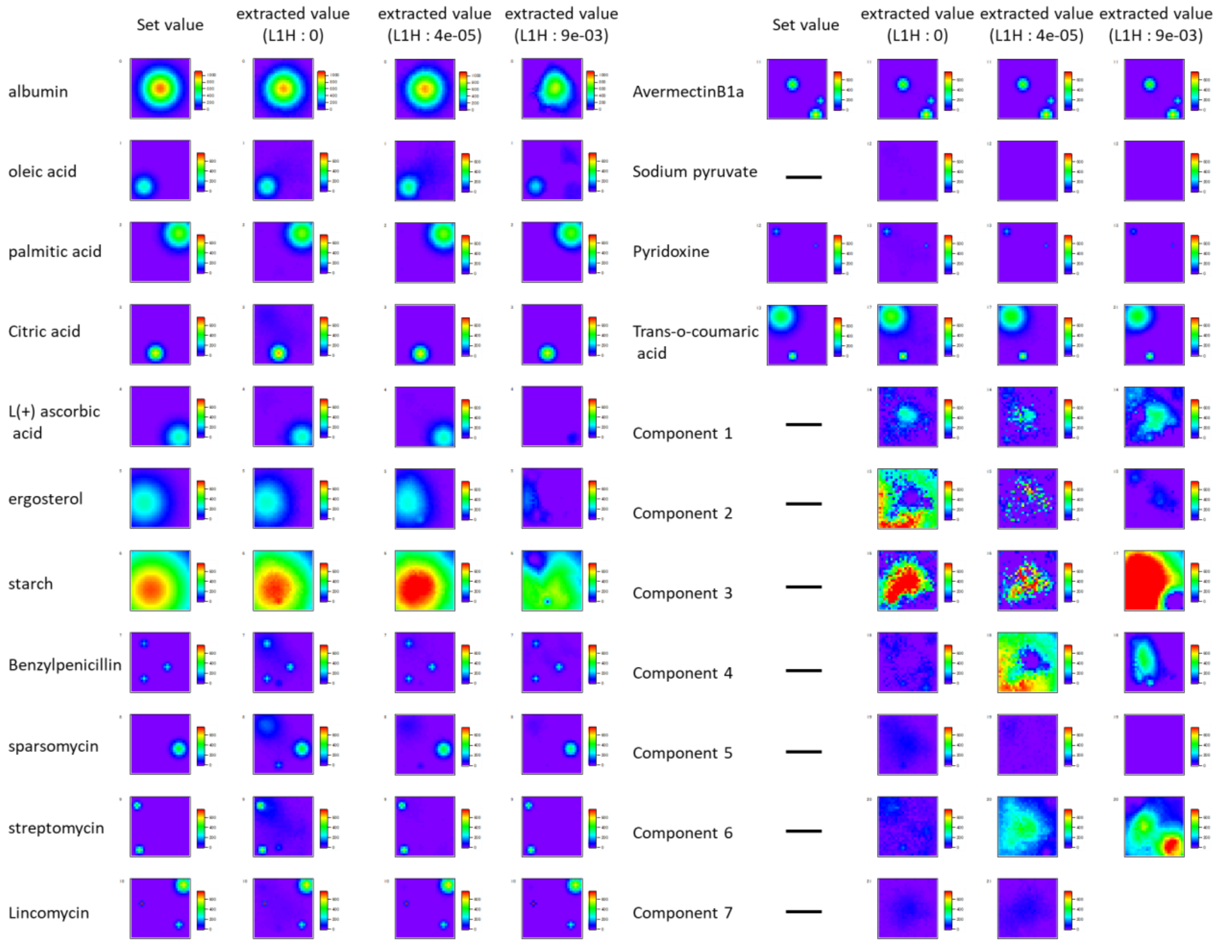

Figure S12. Comparison of Raman spectral intensity profile between set and resolved via MCR-ALS. " – " indicates the component was not used for preparing the dataset.

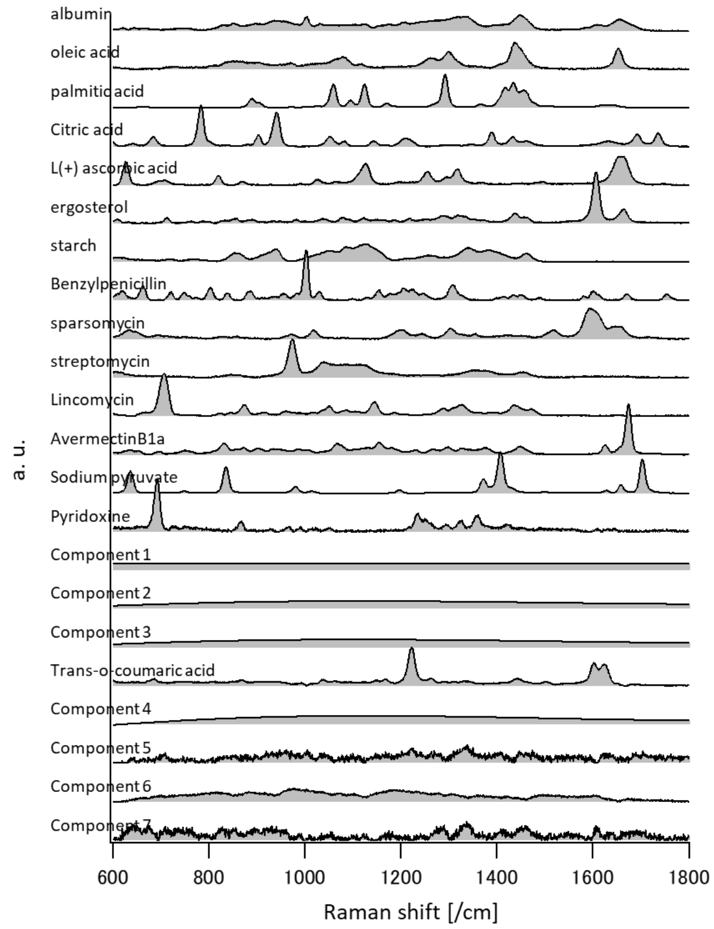

Figure S13. semi-supervised MCR resolved spectra for 14 components Raman spectral dataset with L1H of 4e-05. pyridoxine, trans-o-coumaric acid, and Component 1 – 7 were detected during calculation without reference spectra.

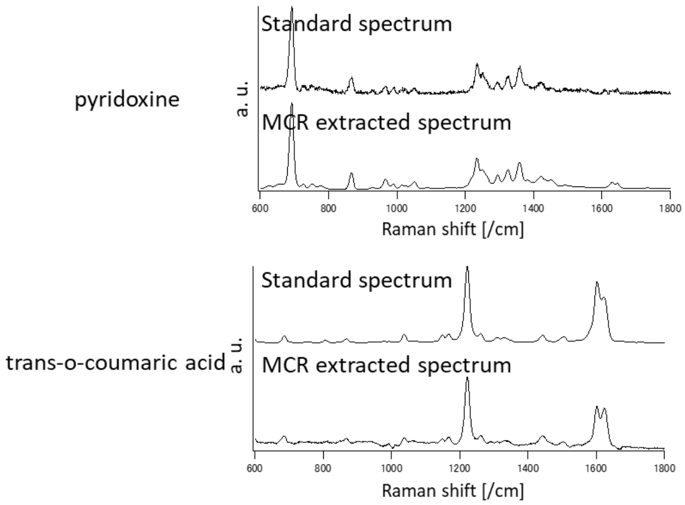

Figure S14. Raman spectra of trans-o-coumaric acid and pyridoxine obtained by semi-supervised MCR-ALS (L1H: 4e-05)

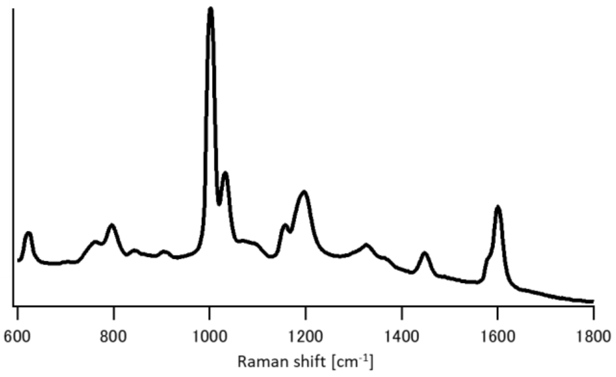

Figure S15. Raman spectra of polystyrene measured from the bottom of a culture dish

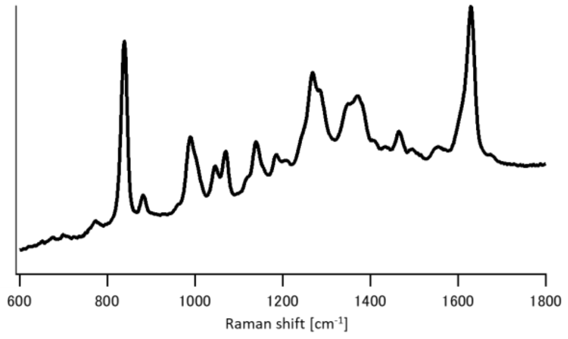

Figure S16. Raman spectra of undecylprodigiosin powder

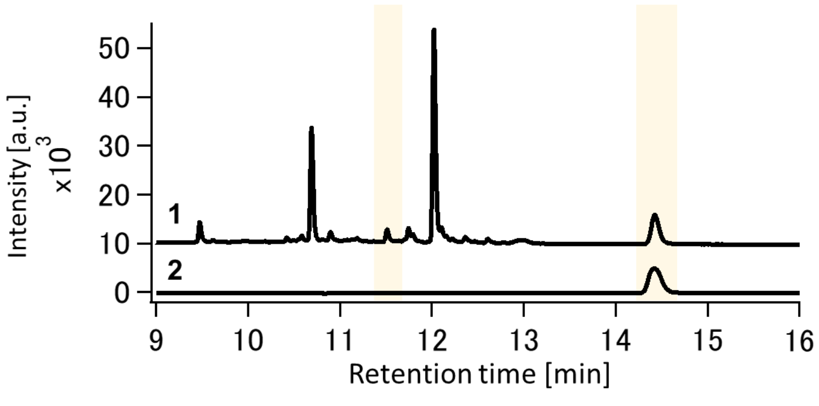

Figure S17. HPLC chromatograms of 1) *S. coelicolor* A3(2) extract (WAP medium, day 9) and 2) undecylprodigiosin dissolved in methanol. UV absorption at 524 nm was detected. Undecylprodigiosin was eluted around 14.5 min with $[M+H]^+$ 394.3 m/z, and actinorhodin was eluted around 11.4 min

with $[M+H]^+$ 635.1 m/z.

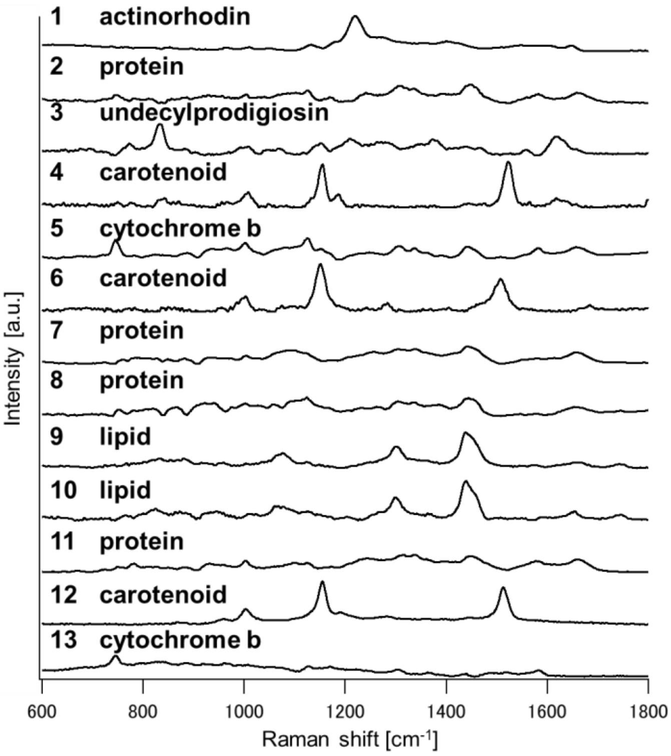

Figure S18. Biomolecular Raman spectra obtained from four actinomycetes Raman spectra resolved by MCR-ALS spectra 1~ 4 were obtained from *S. coelicolor* A3(2), 5 ~ 7 from *Thermomonospora*. sp, 8,9 from *S. thermocarboxydus*, 10 ~ 13 from *Saccharopolyspora.* sp. (1) actinorhodin, (2) protein, (3) undecylprodigiosin, (4) carotenoid, (5) cytochrome b, (6) carotenoid, (7, 8) protein, (9, 10) protein, (11) protein, (12) carotenoid, (13) cytochrome b. The four Raman spectra of protein were averaged when utilized in the screening analysis.

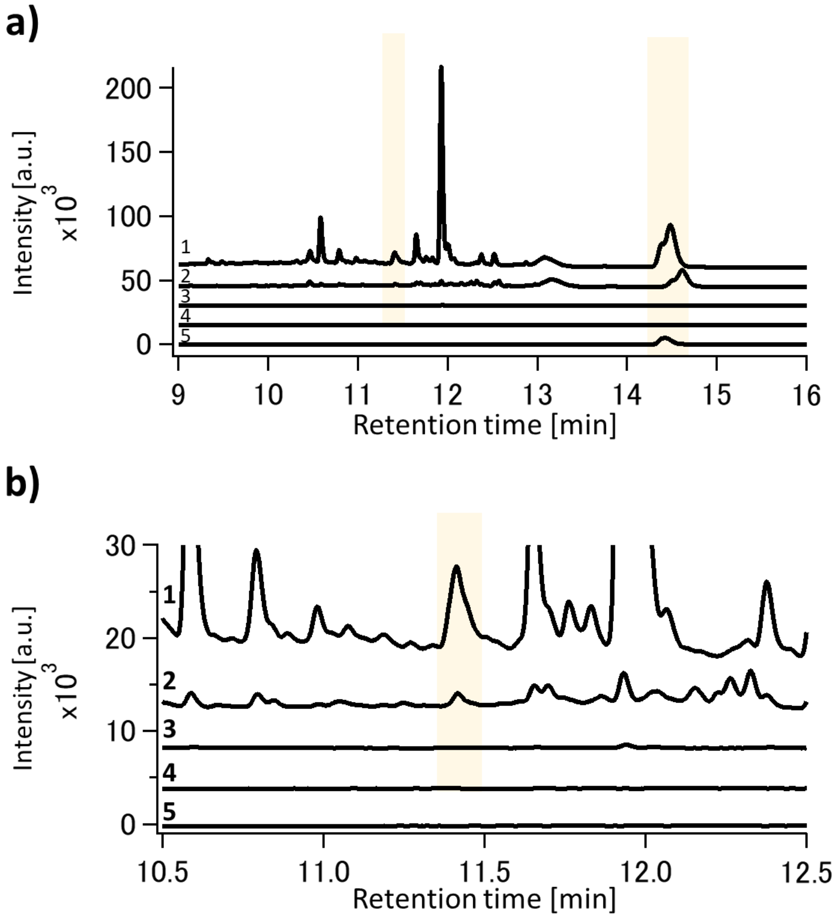

Figure S19. HPLC chromatograms of *S. coelicolor* A3(2) extract with various culture conditions.
UV absorption at 524 nm was detected. Actinorhodin was eluted around 11.4 min with $[M+H]^+$ 635.1 m/z and undecylprodigiosin around 14.5 min with $[M+H]^+$ 394.3 m/z. (a) 1) *S. coelicolor* A3(2) WAP culture extract at day 10, 2) at day 5, 3) at day 2, 4) YD agar culture extract at day 3, and 5) undecylprodigiosin dissolved in MeOH. (b) The expanded chromatograms.

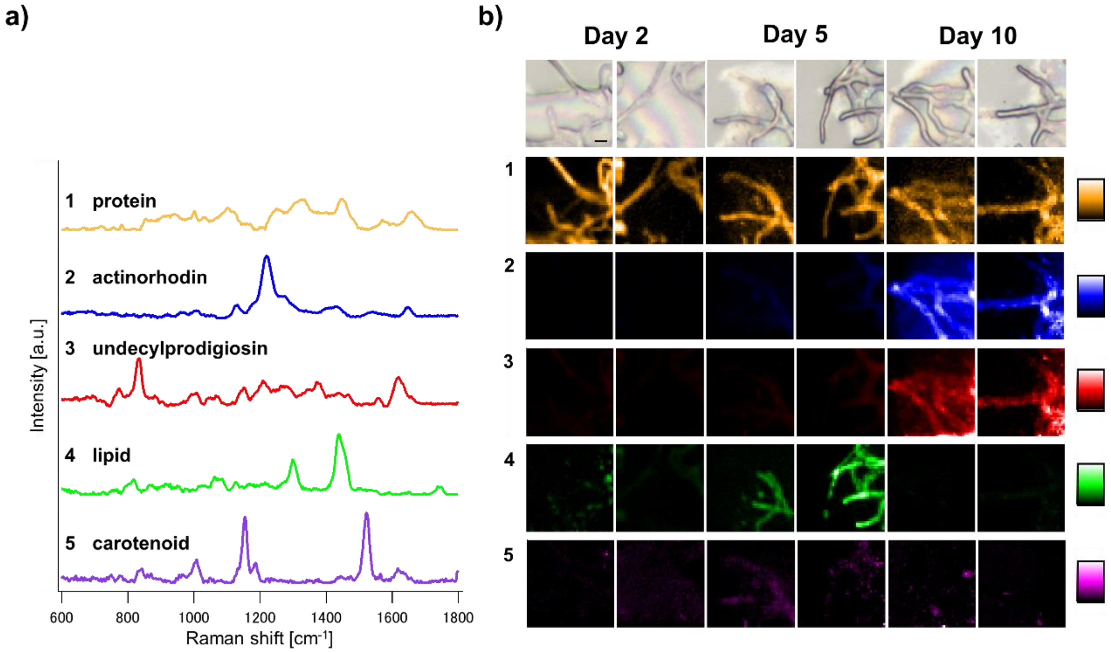

Figure S20. Time course Raman imaging of various biomolecules produced by *S. coelicolor* A3(2) via MCR-ALS with the LASSO constraint α_{L1H}: 5e-05. (a) Biomolecular Raman spectra obtained by MCR-ALS (1) protein, (2) actinorhodin, (3) undecylprodigiosin (4) lipids, (5) carotenoid. (b) Optical microscopic images of mycelia and Raman imaging corresponding to each component. Scale bar = 2 μm

All of the data matrices were combined to one matrix, and MCR-ALS was performed.

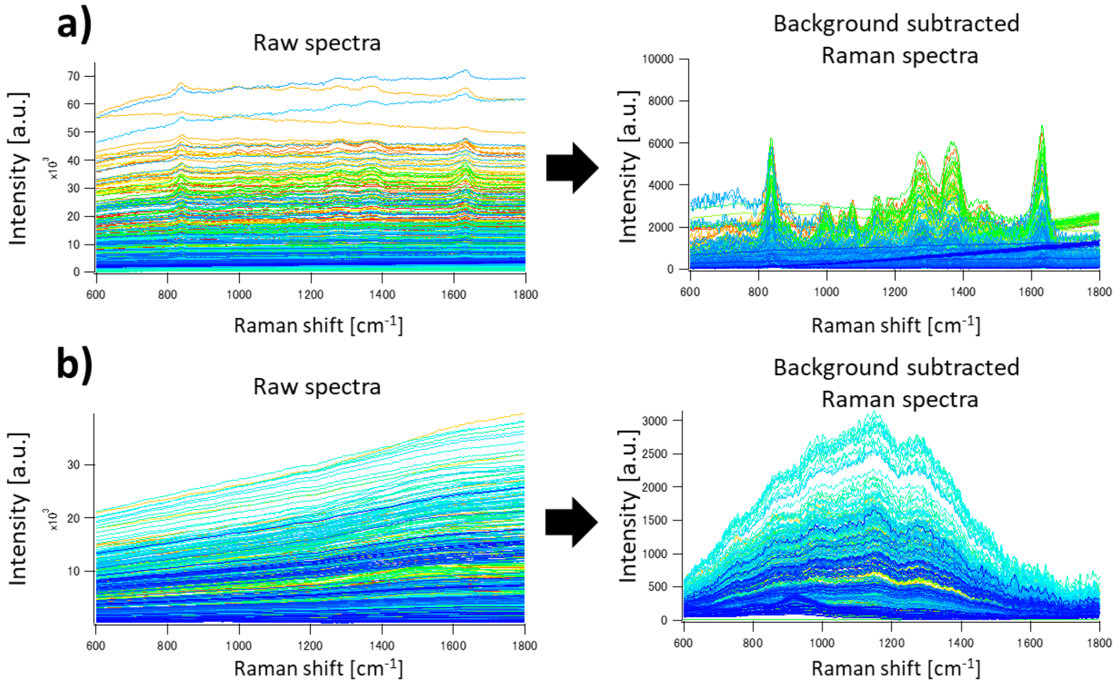

Figure S21. Acquired Raman spectra from actinomycetes colonies. (a) Raman spectra obtained from *S. coelicolor* A3(2) under undecylprodigiosin and actinorhodin producing condition and background subtracted spectra. (b) Raman spectra obtained from *S. nodosus* under AmB producing condition and background subtracted spectra.

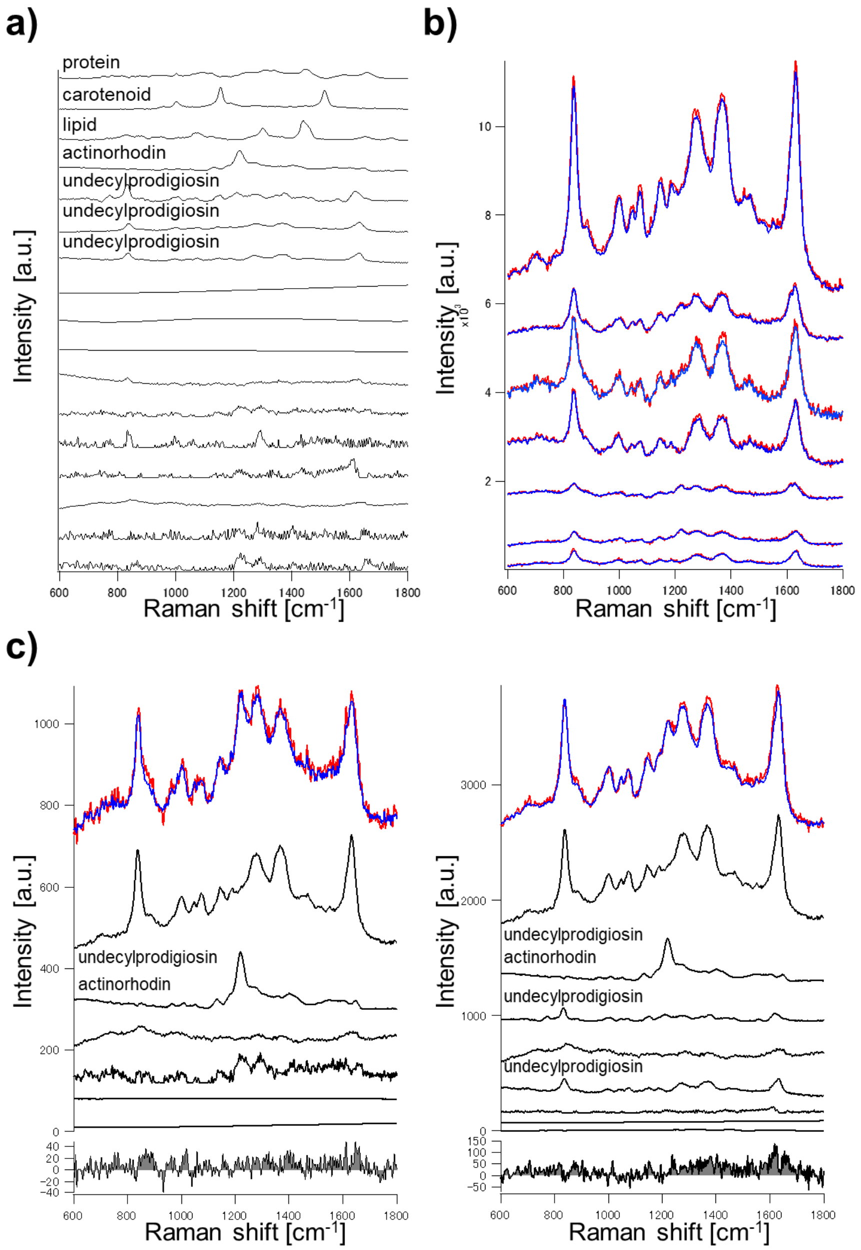

Figure S22. Accuracy of MCR-ALS fitting for Raman spectra from *S. coelicolor* A3(2) colonies under undecylprodigiosin and actinorhodin production with the LASSO constraint α_{L1H}:0.029. (a) MCR components. The unassigned components are regarded as background or noise. The spectral components except for protein, carotenoid, lipid, actinorhodin, and the top undecylprodigiosin were obtained from random value components used for initializing MCR calculations. (b) Acquired Raman spectra (red) and MCR reconstructed spectra (blue). (c) Acquired Raman spectra (red), detected MCR components (black) and residual (gray shading). Protein, carotenoid, and lipid signal was as weak as noise intensity, almost undetectable overall. spectral plots show negligible residual with no obvious Raman bands (dark), which means the MCR analysis accurately explains the Raman spectral feature observed. Undecylprodigiosin was detected by three spectral components, including reference spectrum. It might be due to the difference in the chemical state of the compounds in the measurement, which possibly caused slight spectral differences. The Raman images of undecylprodigiosin in Figure 4 are shown as the sum of these spectral intensities.

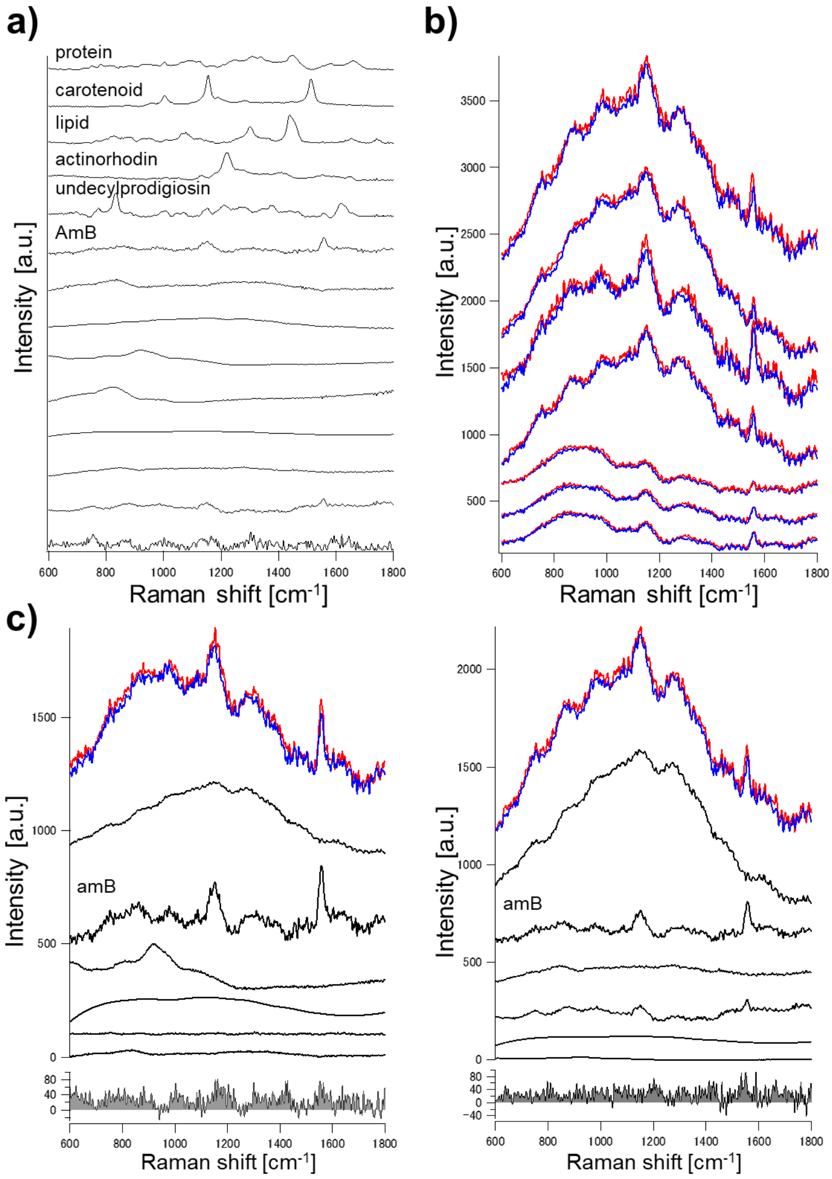

Figure S23. Accuracy of MCR-ALS fitting for Raman spectra from *S. nodosus* colonies under AmB production with the LASSO constraint α_{L1H}: 0.0195. (a) MCR components. The unassigned components are regarded as background or noise. The spectral components except for protein, carotenoid, lipid, actinorhodin, and undecylprodigiosin were obtained from random value components used for initializing MCR calculations. (b) Acquired Raman spectra (red) and MCR reconstructed spectra (blue). (c) Acquired Raman spectra (red), detected MCR components (black) and residual (gray shading). Protein, carotenoid, lipid, undecylprodigiosin and actinorhodin signals were below noise intensity, undetectable level. And spectral plots show negligible residual with no obvious Raman bands (dark), which means the MCR analysis accurately explains the Raman spectral feature observed.

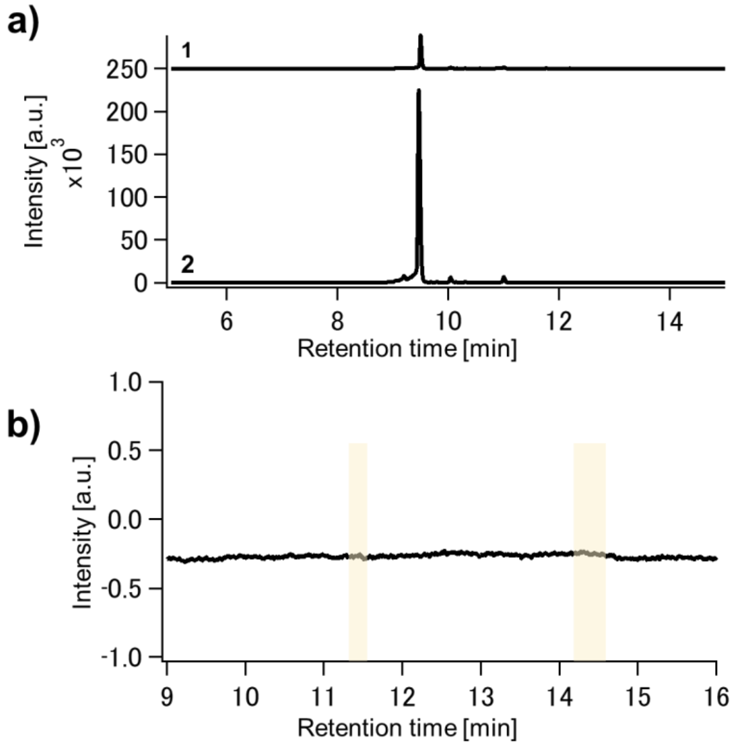

Figure. S24 HPLC chromatograms of *S. nodosus* culture extract. (a) 1: UV absorption at 380 nm was detected. YE starch culture extract 2: AmB dissolved in MeOH. UV absorption at 380 nm were observed around 9.5 min with $[M+H]^+$ 924.5 m/z.(b) UV absorption from *S. nodosus* YE starch culture extract at 524 nm. No UV absorptions related to actinorhodin and undecylprodigiosin were observed.
